# Supplementary material for: Phytochemical Analysis, Antioxidant Activities In Vitro and In Vivo, and Theoretical Calculation of Different Extracts of Euphorbia fischeriana
Source: Molecules. 2023 Jul 2;28(13):5172. doi: 10.3390/molecules28135172 (PMC10343448; doi:10.3390/molecules28135172)
Supplement: Supplementary file 1 [file molecules-28-05172-s001.zip › molecules-2486612-supplementary.pdf]

SUPPLEMENTARY MATERIAL

Phytochemical Analysis, Antioxidant Activities In Vitro and In Vivo, and Theoretical Calculation of Different Extracts of *Euphorbia fischeriana*

Yue Sun [1,2,†], Jia-Xin Feng [1,2,†], Zhong-Bao Wei [3], Hui Sun [2], Li Li [1,2,*], Jun-Yi Zhu [2,4], Guang-Qing Xia [1,2,4,*] and Hao Zang [1,2,4,*]

[1] College of Pharmacy, Yanbian University, Yanji 133000, China; 15981309048@163.com (Y.S.); 13630304082@163.com (J.-X.F.)
[2] Green Medicinal Chemistry Laboratory, School of Pharmacy and Medicine, Tonghua Normal University, Tonghua 134002, China; sunhui9405@163.com (H.S.); swx0527@163.com (J.-Y.Z.)
[3] Institute of Scientific and Technical Information of Jilin, Changchun 130033, China; 13894531050@163.com (Z.-B.W.)
[4] Key Laboratory of Evaluation and Application of Changbai Mountain Biological Gerplasm Resources of Jilin Province, Tonghua 134002, China
* Correspondence: 15044586210@163.com (L.L.); qingguangx@163.com (G.-Q.X.); zanghao_1984@163.com (H.Z.); Tel.: +86-435-320-2678 (L.L. & H.Z.); +86-435-320-8881 (G.-Q.X.)
† These authors contributed equally to this work.

Preparation of preliminary experimental solutions for qualitative phytochemical analysis

Aqueous extraction solutions

Euphorbia fischeriana powder (5 g) was weighed and passed through a sieve (20 mesh). Distilled water (50 mL) was added and the mixture was allowed to stand overnight at room temperature. Next, 5 mL of filtrate was obtained by filtration, and this filtrate was analysed to check for amino acids and proteins. The remaining residue and leaching solution were heated at 60 °C for 10 min. After heating, the mixture was filtered immediately. This filtrate was used to check for carbohydrates, organic acids, saponins, glycosides, phenols, tannins and cyanogenic glycosides.

Methanol extraction solutions

Euphorbia fischeriana powder (5 g) was weighed and passed through a sieve (20 mesh). Ethyl ether (50 mL) was added and the mixture was heated under reflux for 10 min. The filter residue was transferred back into the bottle after filtration. Next, 35 mL of methanol was added and the mixture was heated under reflux for 10 min. After heating, the mixture was filtered immediately. This filtrate was used to check for flavonoids, anthraquinones, cardiac glycosides, coumarins, lactones, volatile oils, terpenoids, steroids, lipids and alkaloids.

Petroleum ether extraction solutions

Euphorbia fischeriana powder (3 g) was weighed and passed through a sieve (20 mesh). Petroleum ether (15 mL) was added and the mixture was allowed to stand at room temperature for 4 h. Next, 5 mL of filtrate was obtained by filtration, and this filtrate was analysed to check for volatile oils, lipids, steroids and triterpenoids.

Qualitative phytochemical analysis

Tests for proteins

Ninhydrin tests

1 mL of aqueous extraction solution was mixed with 1 mL of 0.2% ninhydrin solution. The mixture was boiled for 5 min. Development of a purple colour indicated the presence of amino acids or proteins.

Biuret tests

1 mL of aqueous extraction solution was mixed with 1 mL of solution A (0.1 g/mL NaOH), and then two drops of solution B (0.01 g/mL $CuSO_4$) were added. This mixture was shaken, and a purple, red, or purplish-red colour indicated the presence of amino acids or proteins.

Tests for carbohydrates

Fehling's tests

Equal volumes of solution A (34.66 g of $CuSO_4·5H_2O$ dissolved in 500 mL of distilled water) and solution B (173 g of sodium potassium tartrate tetrahydrate and 50 g of NaOH dissolved in 500 mL of distilled water) were mixed together. A sample (1 mL) of this mixture was then mixed with 1 mL of aqueous extraction solution. The resulting mixture was boiled gently. Formation of a brick-red precipitate indicated the presence of reducing sugars.

Benedict's tests

1 mL of aqueous extraction solution was mixed with 1 mL of Benedict's reagent and the resulting mixture was boiled gently. Formation of a reddish-brown precipitate indicated the presence of carbohydrates.

Molisch's tests

1 mL of aqueous extraction solution was mixed with 1 mL of Molisch's solution (2 g of α-naphthol dissolved in 100 mL of 95% ethanol). The mixture was then poured carefully into another test tube containing 1 mL of H_2SO_4. A purple ring at the aqueous phase/organic phase interface indicated the presence of carbohydrates.

Iodine tests

1 mL of aqueous extraction solution was mixed with 1 mL of iodine solution (127 mg of iodine and 200 mg of KI dissolved in 10 mL of distilled water). Development of a dark blue or purple colour indicated the presence of carbohydrates.

Tests for phenols

$FeCl_3$ tests

1 mL of aqueous extraction solution was mixed with 1 mL of 2% $FeCl_3$ solution. Development of a blue-green or black colour indicated the presence of phenols.

$FeCl_3$-$K_3[Fe(CN)_6]$ tests

A few drops of aqueous extraction solution were added onto a thin-layer chromatography plate, and the chromogenic reagent (1% $K_3[Fe(CN)_6]$ solution was mixed with 2% $FeCl_3$ solution in equal volumes) was sprayed onto the plate, thus generating a blue colour. Then, 2 M HCl was sprayed onto the plate, and a darker colour indicated the presence of phenols.

Diazotization tests

1 mL of aqueous extraction solution was mixed with 1 mL of 3% Na_2CO_3 solution. The resulting mixture was boiled for 3 min and then cooled in ice water. Two drops of newly prepared diazotization reagent were added. Development of a red colour indicated the presence of phenols.

Tests for organic acids

pH tests

The pH of aqueous extraction solution was measured with pH meter. A pH value below 7.0 indicated the presence of organic acids.

Blue litmus paper tests

A few drops of aqueous extraction solution were placed on a blue litmus paper. Development of a red colour indicated the presence of organic acids.

Bromocresol green tests

A few drops of aqueous extraction solution were added onto a thin-layer chromatography plate. Chromogenic reagent (0.1 g of bromocresol green dissolved in 500 mL of ethanol and mixed with 5 mL of 0.1 N NaOH) was sprayed onto the plate. Development of a yellow colour on a blue background indicated the presence of organic acids.

Tests for tannins

$FeCl_3$ tests

The experimental procedure was the same as that described in $FeCl_3$ tests. Development of a blue-green or black colour indicated the presence of tannins.

Bromine water tests

Bromine water (3%) was added to 1 mL of aqueous extraction solution. Formation of a precipitate indicated the presence of tannins.

Lead acetate tests

1 mL of lead acetate solution was added to 1 mL of aqueous extraction solution; a precipitate was considered evidence for the presence of tannins.

Lime water tests

Clear lime water (1 mL) was added to 1 mL of aqueous extraction solution. Formation of a precipitate indicated the presence of tannins.

Gelatin tests

1 mL of aqueous extraction solution was mixed with 1 mL of 0.5% gelatin dissolved in 10% NaCl solution. Turbidity indicated the presence of tannins.

Tests for flavonoids

Shinoda tests

An appropriate amount of magnesium powder was added to 1 mL of methanol extraction solution, followed by two drops of HCl. Development of a red to red-purple colour indicated the presence of flavonoids.

Alkaline reagent tests

1 mL of methanol extraction solution was mixed with 1 mL of 2% NaOH solution. Development of an intense yellow colour followed by a change to colourless on addition of a few drops of diluted HCl indicated the presence of flavonoids.

$AlCl_3$ tests

A few drops of methanol extraction solution were added onto a thin-layer chromatography plate. A 1% $AlCl_3$ methanol solution was sprayed onto the plate. Observation of yellow-green fluorescence under an ultraviolet lamp indicated the presence of flavonoids.

Lead acetate test

A few drops of lead acetate solution were added to 1 mL of methanol extraction solution. Formation of a yellow precipitate indicated the presence of flavonoids.

Tests for saponins

Foam tests

1 mL of aqueous extraction solution was mixed with 5 mL of distilled water. This mixture was shaken and then left to stand for 10 min. Formation of a stable foam indicated the presence of saponins.

Tests for steroids and triterpenoids

Liebermann-Burchard tests

5 mL of the aqueous extraction solution was placed in an evaporating dish and then evaporated. The residue was dissolved in 1 mL of acetic anhydride. One drop of H_2SO_4 was added and development of a red or purple colour indicated the presence of triterpenoids. Development of a blue-green colour indicated the presence of steroids.

Salkowski tests

1 mL of methanol extraction solution was mixed with 1 mL of $CHCl_3$. Then, 1 mL of H_2SO_4 was added carefully, and the mixture was shaken gently. A reddish-brown colour in the $CHCl_3$ layer and green fluorescence in the H_2SO_4 layer indicated the presence of steroids or triterpenoids.

Tests for terpenoids

$CHCl_3$-H_2SO_4 tests

1 mL of methanol extraction solution was mixed with 2 mL of $CHCl_3$ and then evaporated. H_2SO_4 (2 mL) was added carefully, and the mixture was heated at 60 °C for 2 min. Development of a grey colour indicated the presence of terpenoids.

Vanillin-H_2SO_4 tests

A few drops of petroleum ether extraction solution were added onto a thin-layer chromatography plate. Chromogenic reagent was prepared by dissolving 5 g of vanillin in 100 mL of 10% H_2SO_4 ethanol solution, and then sprayed onto the plate. Development of a red, blue, or purple colour indicated the presence of volatile oils, terpenoids, and steroids.

Tests for alkaloids

Bertrad's reagent tests

1 mL of ethanol extraction solution was mixed with 1 mL of tungstosilicic acid reagent. The reagent was prepared by dissolving 5 g of tungstosilicic acid hydrate in 100 mL of distilled water and adding a small amount of HCl to adjust the pH to 2.0. Formation of a pale yellow or off-white precipitate indicated the presence of alkaloids.

Dragendorff's reagent tests

1 mL of ethanol extraction solution was mixed with 1 mL of Dragendorff 's reagent. For the reagent, solution A (850 mg of bismuth subnitrate dissolved in 40 mL of distilled water and 10 mL of acetic acid) and solution B (8 g of KI dissolved in 20 mL of distilled water) were mixed in equal volumes to prepare a stock solution. A sample of this stock solution (10 mL) was then mixed with 20 mL of acetic acid and diluted to 100 mL with distilled water. Formation of a light yellow or reddish brown precipitate indicated the presence of alkaloids.

Mayer's reagent tests

1 mL of ethanol extraction solution was mixed with 1 mL of Mayer's reagent. For the reagent, solution A (1358 mg of $HgCl_2$ dissolved in 60 mL of distilled water) and solution B (5 g of KI dissolved in 10 mL of distilled water) were mixed and then diluted to 100 mL with distilled water. Formation of a white or light yellow precipitate indicated the presence of alkaloids.

Tests for anthraquinones

Borntrager's tests

1 mL of methanol extraction solution was mixed with 1 mL of 10% NaOH solution. A red colour developed. Next, a small volume of 30% H_2O_2 solution was added and the mixture was heated at 60 °C. HCl solution was then added and the red colour disappeared, finally, NaOH solution was added and development of a red colour indicated the presence of anthraquinones.

Magnesium acetate tests

Three drops of 1% magnesium acetate methanol solution were added to 1 mL of methanol extraction solution. Development of a red colour indicated the presence of anthraquinones.

Tests for coumarins and lactones

Hydroxamic acid iron tests

Three drops of 7% hydroxylamine hydrochloride methanol solution and 10% KOH methanol solution were added to 1 mL of methanol extraction solution. After heating at 60 °C, 5% HCl was added to adjust the pH to 3.0-4.0. Next, two drops of 1% $FeCl_3$ ethanol solution were added. Development of a orange or purple colour indicated the presence of coumarins and lactones.

Diazotization tests

Methanol extraction solution was used. The experimental procedure was the same as that described in

Diazotization tests. Development of a red colour indicated the presence of coumarins and lactones.

Fluorescence tests

A few drops of methanol extraction solution were added onto a thin-layer chromatography plate and blue-green fluorescence was observed under ultraviolet lamp. 1% KOH solution was sprayed onto the plate. Generation of intense fluorescence indicated the presence of coumarins.

Tests for volatile oils and fats

Phosphomolybdic acid tests

A few drops of petroleum ether extraction solution were added onto a thin-layer chromatography plate and 25% phosphomolybdic acid solution (2.5 g of phosphomolybdic acid hydrate dissolved in 10 mL of absolute ethanol) was sprayed onto the plate. Development of a blue colour indicated the presence of lipids, triterpenoids, and steroids.

Vanillin-H_2SO_4 tests

Methanol extraction solution was used. The experimental procedure was the same as that described in Vanillin-H_2SO_4 tests. Development of a red, blue, or purple colour indicated the presence of volatile oils, terpenoids, and steroids.

Sudan tests

One drop of Sudan III solution (0.1 g of sudan III dissolved in 10 mL of 95% ethanol) was added to 1 mL of methanol extraction solution. Development of an orange colour indicated the presence of oils and fats. One drop of Sudan IV solution (0.01 g of Sudan IV dissolved in 5 mL of acetone, followed by addition of 5 mL of 70% ethanol) was added to 1 mL of methanol extraction solution. Development of a red colour indicated the presence of oils and fats.

Tests for cardiac glycosides

Kedde tests

A few drops of methanol extraction solution were added onto a thin-layer chromatography plate. Chromogenic reagent was prepared by mixing solution A (2% methanol solution of 3,5-dinitrobenzoic acid) and solution B (2 M KOH solution) in equal volumes. The reagent was sprayed onto the plate. Development of a purple-red colour followed by a change to colourless indicated the presence of cardiac glycosides.

Raymond tests

Methanol extract (1 mg) was dissolved in 50% ethanol. Both 2% *m*-dinitrobenzene ethanol solution (0.1 mL) and 20% NaOH solution (0.2 mL) were added. Development of a blue-purple colour indicated the presence of cardiac glycosides.

Legal tests

Methanol extract (1 mg) was dissolved in two drops of pyridine. One drop of 3% sodium nitroprusside solution and one drop of 2 M NaOH solution were added. Development of a dark red colour followed by a change to colourless indicated the presence of cardiac glycosides.

Tests for cyanogenic glycosides

Prussian blue tests

1 g of TL powder was placed in a test tube, 2 mL of distilled water was added, and the test tube was immediately wrapped with filter paper. Then, one drop of 10% KOH solution was added onto the filter paper, and the system was heated at 60 °C for 30 min. Next, one drop each of 10% ferrous sulphate, 10% HCl, and 5% $FeCl_3$ were sequentially added onto the filter paper. A blue colour on the filter paper indicated the presence of cyanogenic glycosides.

Quantitative phytochemical analysis

Determination of total carbohydrate content (TCC)

Briefly, 250 μL of *Euphorbia fischeriana* extract in distilled water, 125 μL of phenol solution (5%), and 625 μL of H_2SO_4 were mixed in an Eppendorf tube and incubated for 30 min. Subsequently, 200 μL of the sample was pipetted from each Eppendorf tube onto a microplate. A calibration curve was produced based on glucose (0–200 mg/L) as a standard. The absorbance of the sample was recorded at 490 nm against a blank

sample consisting of *Euphorbia fischeriana* extract with distilled water. The mean of three readings was used and TCC was expressed in milligrams of glucose equivalents (GE)/g of *Euphorbia fischeriana* extract.

Determination of total protein content ($TP_{ro}C$)

Briefly, 200 µL of bicinchoninic acid (BCA) working solution and 20 µL of *Euphorbia fischeriana* extract in distilled water were mixed in a microplate and incubated at 37 °C for 30 min. A calibration curve was produced based on bovine serum albumin (BSA) (0–500 mg/L) as a standard. The absorbance of the sample was recorded at 562 nm against a blank sample consisting of *Euphorbia fischeriana* extract with distilled water. The mean of three readings was used and $TP_{ro}C$ was expressed in milligrams of BSA equivalents (BSAE)/g of *Euphorbia fischeriana* extract.

Determination of total triterpenoid content ($TT_{ri}C$)

Briefly, 180 µL of *Euphorbia fischeriana* extract in acetic anhydride and 20 µL of H_2SO_4 were mixed in a microplate and incubated at room temperature for 10 min. A calibration curve was produced based on ginsenoside Re (0–400 mg/L) as a standard. The absorbance of the sample was recorded at 350 nm against a blank sample consisting of *Euphorbia fischeriana* extract with acetic anhydride. The mean of three readings was used and $TT_{ri}C$ was expressed in milligrams of ginsenoside Re equivalents (GRE)/g of *Euphorbia fischeriana* extract.

Determination of total phenolic content ($TP_{he}C$)

Briefly, 100 µL of Folin & Ciocalteu's phenol reagent (FC reagent) (1 M) and 200 µL of *Euphorbia fischeriana* extract in distilled water were mixed in an Eppendorf tube and incubated for 5 min. Subsequently, 500 µL of Na_2CO_3 solution (20%) was added and allowed to stand at room temperature for 40 min in the dark (with mixing every 10 min). Subsequently, 200 µL of the sample was pipetted from each Eppendorf tube onto a microplate. A calibration curve was produced based on gallic acid (0–100 mg/L) as a standard. The absorbance of the sample was recorded at 750 nm against a blank sample consisting of *Euphorbia fischeriana* extract with distilled water and Na_2CO_3. The mean of three readings was used and $TP_{he}C$ was expressed in milligrams of gallic acid equivalents (GAE)/g of *Euphorbia fischeriana* extract.

Determination of total flavonoid content (TFC)

Briefly, 100 µL of $AlCl_3$ (2%) in methanol and 100 µL of *Euphorbia fischeriana* extract in methanol were mixed in a microplate and incubated at room temperature for 10 min. A calibration curve was produced based on quercetin (0–100 mg/L) as a standard. The absorbance of the sample was recorded at 415 nm against a blank sample consisting of *Euphorbia fischeriana* extract with methanol. The mean of three readings was used and TFC was expressed in milligrams of quercetin equivalents (QE)/g of *Euphorbia fischeriana* extract.

Determination of total tannin content ($TT_{an}C$)

Briefly, 200 µL of FC reagent (1 M) and 200 µL of *Euphorbia fischeriana* extract in distilled water were mixed in an Eppendorf tube and incubated for 5 min. Subsequently, 100 µL of Na_2CO_3 solution (20%) and 1500 µL of distilled water were added and allowed to stand at room temperature for 30 min in the dark (with mixing every 10 min). Subsequently, 200 µL of the sample was pipetted from each Eppendorf tube onto a microplate. A calibration curve was produced based on tannic acid (0–200 mg/L) as a standard. The absorbance of the sample was recorded at 725 nm against a blank sample consisting of *Euphorbia fischeriana* extract with distilled water and Na_2CO_3. The mean of three readings was used and $TT_{an}C$ was expressed in milligrams of tannic acid equivalents (TAE)/g of *Euphorbia fischeriana* extract.

Determination of gallotannin content (GC)

Briefly, 875 µL of *Euphorbia fischeriana* extract in methanol and 375 µL of saturated KIO_3 solution were mixed in an Eppendorf tube and incubated at 15 °C for 120 min. A calibration curve was produced based on gallic acid (0–400 mg/L) as a standard. The absorbance of the sample was recorded at 550 nm against a blank sample (KIO_3 was replaced with distilled water). The mean of three readings was used and GC was expressed in milligrams of gallic acid equivalents (GAE)/g of *Euphorbia fischeriana* extract.

Determination of condensed tannin content (CTC)

Briefly, 4 mg of phloroglucinol was added to 2 mL of *Euphorbia fischeriana* extract in distilled water. Subsequently, 1 mL of HCl solution and 1 mL of formaldehyde solution were added and mixed in an Eppendorf tube and incubated at room temperature overnight. The precipitate was separated by filtration, the unprecipitated phenolics were measured in the filtrate according to the method of $TP_{he}C$.

Determination of total alkaloid content (TAC)

Berberine hydrochloride (1.24–12.36 mg/L) was used as a reference material to construct a standard curve. The absorbance was obtained at 420 nm against a blank sample of chloroform. The mean of three measurements was calculated. The total alkaloid content is expressed in milligrams of berberine hydrochloride equivalents per gram of *Euphorbia fischeriana* extract.

Antioxidant activity assay

DPPH assay

Briefly, 100 μL of *Euphorbia fischeriana* extract in methanol and 100 μL of DPPH in methanol (50 μM) were mixed in a microplate and allowed to stand at room temperature for 20 min in the dark. The absorbance of the sample was recorded at 515 nm. Trolox was used as positive reference and its standard curve is y=11.595x+0.1821. The Half-maximal inhibitory concentration (IC_{50}) values were calculated and expressed as the mean ± standard deviation (SD) in μg/mL.

ABTS assay

Briefly, 190 μL of diluted ABTS solution and 10 μL of *Euphorbia fischeriana* extract in DMSO were mixed in a microplate and incubated for 20 min in the dark. The absorbance of the sample was recorded at 734 nm. Trolox was used as positive reference and its standard curve is y=0.8026x+11.878. The IC_{50} values were calculated and expressed as the mean ± SD in μg/mL.

Hydroxyl radical assay

Briefly, 50 μL of *Euphorbia fischeriana* extract in DMSO, 50 μL of $FeSO_4$ solution (3 mM) and 50 μL of H_2O_2 solution (3 mM) were mixed in a microplate and incubated for 10 min. After then 50 μL of salicylic acid solution (6 mM) was added and incubated at room temperature for 30 min in the dark. The absorbance of the sample was recorded at 492 nm. Trolox was used as positive reference and its standard curve is y=0.0327x+35.047. The IC_{50} values were calculated and expressed as the mean ± SD in μg/mL.

Superoxide radical assay

Briefly, 45 μL of *Euphorbia fischeriana* extract in DMSO (10 mg/mL), 15 μL of *p*-nitroblue tetrazolium chloride (NBT) in DMSO (1 mg/mL) and 150 μL of NaOH in DMSO (50 μM) were mixed in a microplate and the absorbance of the sample was recorded immediately at 560 nm against a blank sample (NBT was replaced with DMSO). Curcumin was used as a positive reference and its standard curve is y=84.38239x/122.02931+x. The scavenging activity was expressed as % scavenging rate and was calculated as follows:

$$\%scavenging = \left(1 - \frac{\Delta A_{sample}}{\Delta A_{control}}\right) \times 100\%$$

FRAP assay

Briefly, 20 μL of *Euphorbia fischeriana* extract in DMSO and 180 μL of FRAP reagent were mixed in a microplate and incubated at 37 °C for 30 min in the dark. A calibration curve was produced based on $FeSO_4$ (0–600 mg/L) as a standard. The absorbance of the sample was recorded at 595 nm. The standard curve of ferrous ion is y=4.416x+0.087. Trolox was used as positive reference. The FRAP was expressed as the Trolox Equivalent Antioxidant Capacity ($TEAC_{FRAP}$).

CUPRAC assay

Briefly, 20 μL of $CuCl_2$ solution (100 mM), 50 μL of neocuproine in 96% ethanol (7.5 mM), 50 μL of NH_4Ac solution, 20 μL of *Euphorbia fischeriana* extract in DMSO, and 30 μL of distilled water were mixed in a microplate and incubated at 50 °C for 20 min. This mixture was allowed to stand at room temperature for 10 min. The absorbance of the sample was recorded at 450 nm. Trolox was used as positive reference and its

standard curve is y=2.8264x+0.0462. The CUPRAC was expressed as the Trolox Equivalent Antioxidant Capacity ($TEAC_{CUPRAC}$).

Iron chelating assay

Briefly, 50 μL of *Euphorbia fischeriana* extract in methanol, 110 μL of ultra-pure water, and 20 μL of $FeCl_2$ solution (0.5 mM) were mixed in a microplate and incubated for 5 min. Subsequently, 20 μL of ferrozine solution (2.5 mM) was added and incubated for 10 min. The absorbance was recorded at 562 nm against a blank sample (ferrozine solution was replaced with water). Ethylenediaminetetraacetic acid disodium salt ($EDTANa_2$) was used as a positive reference and its standard curve is y=2142.2x+26.6. The IC_{50} values were calculated and expressed as the mean ± SD in μg/mL.

Copper chelating assay

Briefly, 40 μL of *Euphorbia fischeriana* extract in ultra-pure water, 140 μL of acetic acid-sodium acetate buffer solution (pH 6.0, 50 mM), and 10 μL of $CuSO_4$ solution (5 mM) were mixed in a microplate and incubated for 30 min. Subsequently, 10 μL of pyrocatechol violet solution (4 mM) was added and incubated for 30 min. The absorbance was recorded at 632 nm against a blank sample (pyrocatechol violet was replaced with water). $EDTANa_2$ was used as a positive reference and its standard curve is y=214.5x+6.9643. The IC_{50} values were calculated and expressed as the mean ± SD in μg/mL.

H_2O_2 assay

Briefly, 70 μL of phenol solution (pH 7.0, 12 mM, in 84 mM phosphate buffer (PBS)), 20 μL of 4-aminoantipyrine solution (pH 7.0, 0.5 mM, in 84 mM PBS), 32 μL of H_2O_2 solution (pH 7.0, 0.7 mM, in 84 mM PBS), 8 μL of horseradish peroxidise (EC 1.11.1.7) solution (pH 7.0, 1 U/mL, in 84 mM PBS) and 70 μL of *Euphorbia fischeriana* extract (pH 7.0, in 84 mM PBS) were mixed in a microplate and the absorbance of the sample was recorded immediately at 504 nm against a blank sample (phenol solution was replaced with PBS). Gallic acid was used as a positive reference and its standard curve is y=0.3898x+25.193. The IC_{50} values were calculated and expressed as the mean ± SD in μg/mL.

Singlet oxygen assay

Briefly, 40 μL of *Euphorbia fischeriana* extract (pH 7.4, in 45 mM PBS), 50 μL of N,N-Dimethyl-4-nitrosoaniline (pH 7.4, 0.2 mM, in 45 mM PBS), 20 μL of histidine solution (pH 7.4, 0.1 mM, in 45 mM PBS), 20 μL of NaClO solution (pH 7.4, 0.1 mM, in 45 mM PBS), 20 μL of H_2O_2 (pH 7.4, 0.1 mM, in 45 mM PBS) and 50 μL of PBS (pH 7.4, 45 mM) were mixed in a microplate and allowed to stand at room temperature for 40 min. The absorbance of the sample was recorded at 440 nm against a blank sample (*Euphorbia fischeriana* extract was replaced with PBS). Ferulic acid was used as a positive reference and its standard curve is y=41.354-42.868. The IC_{50} values were calculated and expressed as the mean ± SD in μg/mL.

HClO assay

HClO was freshly prepared by adjusting the pH of a 1% (v/v) of NaClO to 6.2 with 1% H_2SO_4. The concentration of HClO was determined by reading the absorbance at 235 nm and using the molar extinction coefficient of 100 M^{-1} cm^{-1}. Briefly, twenty-microliters of AR extract aqueous solution, 20 μL of 150 mM taurine aqueous solution, 20 μL of 0.5 mM HClO solution and 140 μL of PBS (pH 7.4, 50 mM) were mixed in a microplate and incubated for 10 min. Subsequently, 2 μL of 2 M KI aqueous solution was added and mixed. The absorbance was recorded at 350 nm against a blank sample (taurine and HClO were replaced with water). Trolox was used as positive reference and its standard curve is y=0.3007x+11.474. IC_{50} values were calculated and expressed as the mean ± SD in μg/mL.

β-Carotene bleaching assay

Briefly, *β*-carotene solution was prepared by dissolving *β*-carotene (2 mg) in $CHCl_3$ (10 mL). Then, 2 mL of the solution was pipetted into a flask and vortex-mixed with linoleic acid (40 mg) and Tween 40 (400 mg). After the removal of $CHCl_3$, 100 mL of oxygenated ultrapure water was added, and the emulsion shaken vigorously. Aliquots (2.4 mL) of the emulsion were pipetted into different test tubes containing 0.1 mL of *Euphorbia fischeriana* extract in methanol (5 mg/mL). BHT and butyl hydroxyanisole (BHA) were used as positive controls. In the control group, *Euphorbia fischeriana* extract was replaced with water. When the

sample was added to the emulsion, it was recorded as t= 0 min. The tubes were capped and placed in a water bath at 60 °C. The absorbance was recorded at 470 nm every 15 min until 120 min. Antioxidant activity coefficient (AAC) was calculated according to the following equation:

$$AAC = \frac{A_{A(120)} - A_{C(120)}}{A_{C(0)} - A_{C(120)}} \times 1000$$

where $A_{A(120)}$ is the absorbance of the antioxidant at 120 min, $A_{C(120)}$ is the absorbance of the control at 120 min, and $A_{C(0)}$ is the absorbance of the control at 0 min.

NO assay

Briefly, 3 mL of *Euphorbia fischeriana* extract in methanol (1 mg/mL) and 3 mL of sodium nitroprusside solution (pH 7.4, 5 mM, in 0.1 M PBS) were mixed in an Eppendorf tube and incubated at 25 °C for 150 min. At intervals, 100 μL of the sample was pipetted from each Eppendorf tube onto a microplate containing 100 μL of Griess reagent. In the control group, *Euphorbia fischeriana* extract was replaced with methanol. The absorbance was recorded at 546 nm against a blank sample (Griess reagent was replaced with distilled water). Curcumin (0.1 mg/mL) was used as a positive reference.

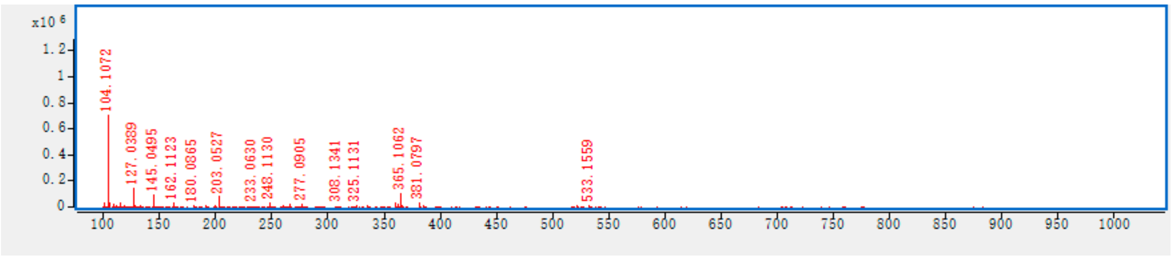

Figure S1 MS spectrum of peak 1

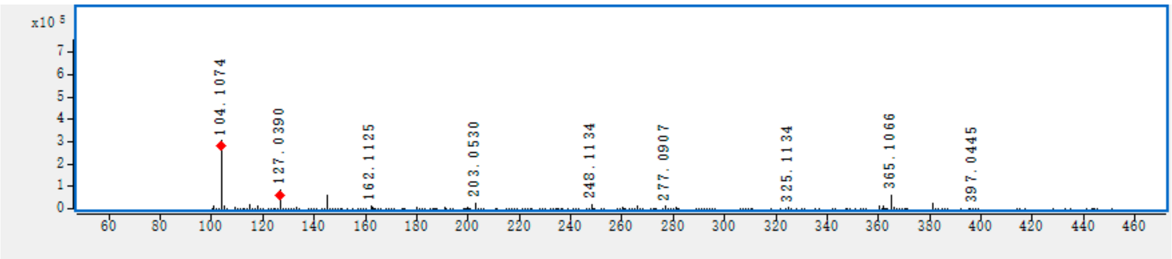

Figure S2 MS/MS spectrum of peak 1

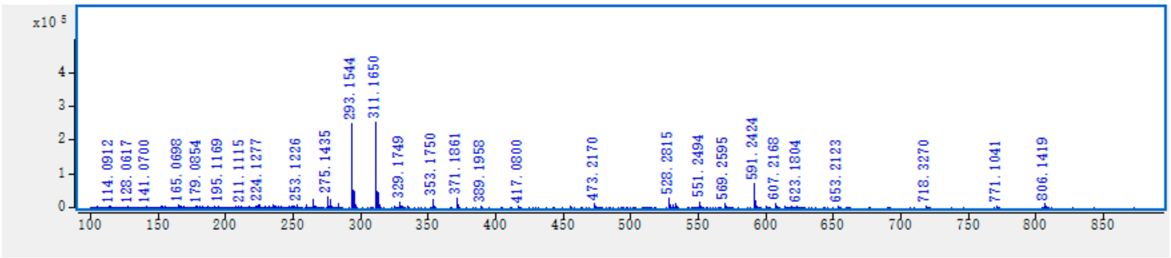

Figure S3 MS spectrum of peak 2

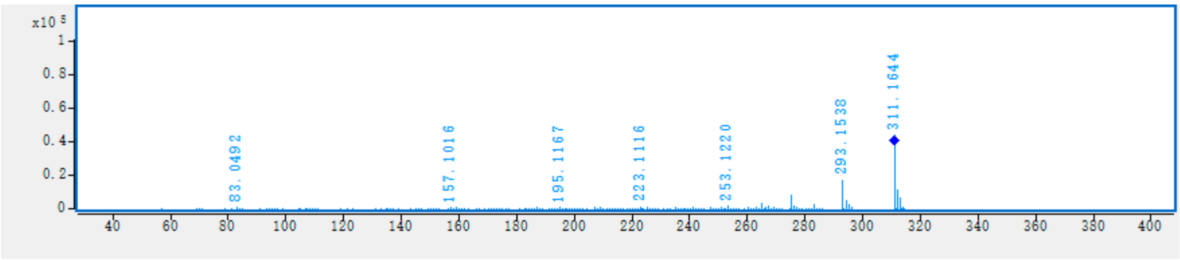

Figure S4 MS/MS spectrum of peak 2

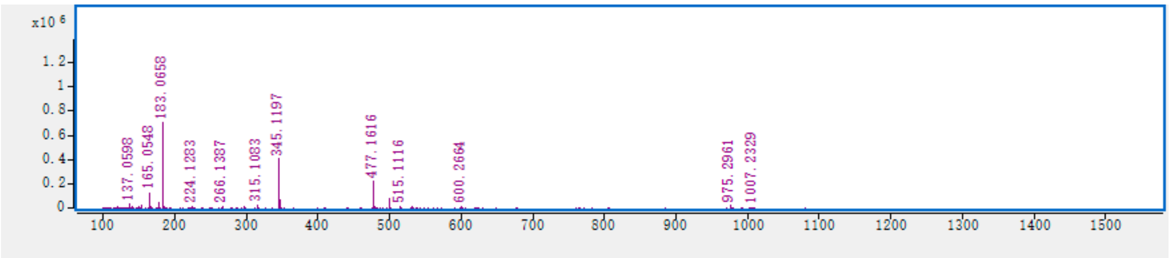

Figure S5 MS spectrum of peak 3

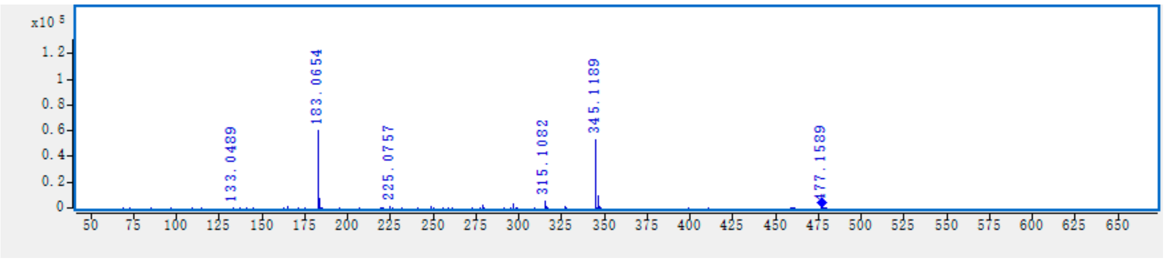

Figure S6 MS/MS spectrum of peak 3

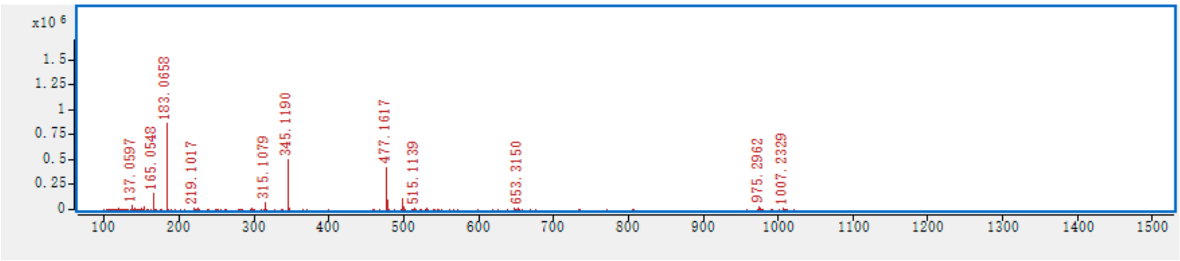

Figure S7 MS spectrum of peak 4

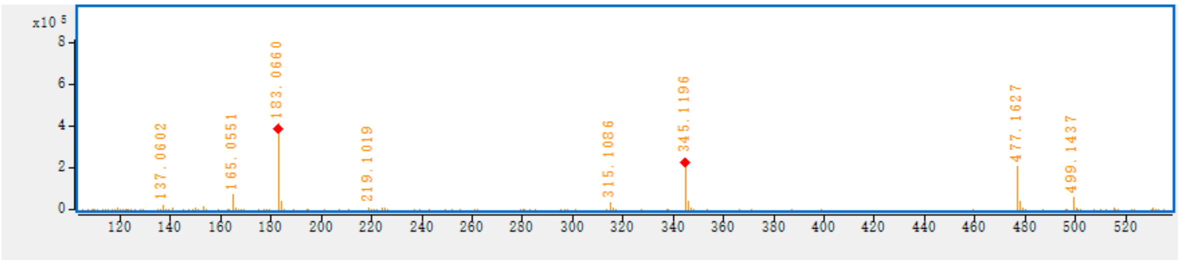

Figure S8 MS/MS spectrum of peak 4

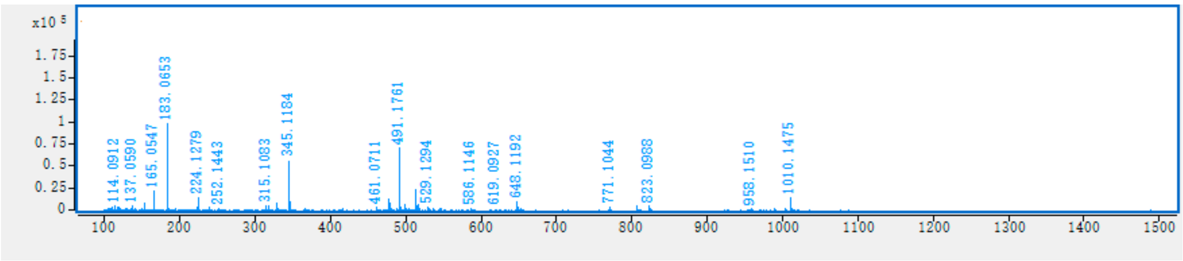

Figure S9 MS spectrum of peak 5

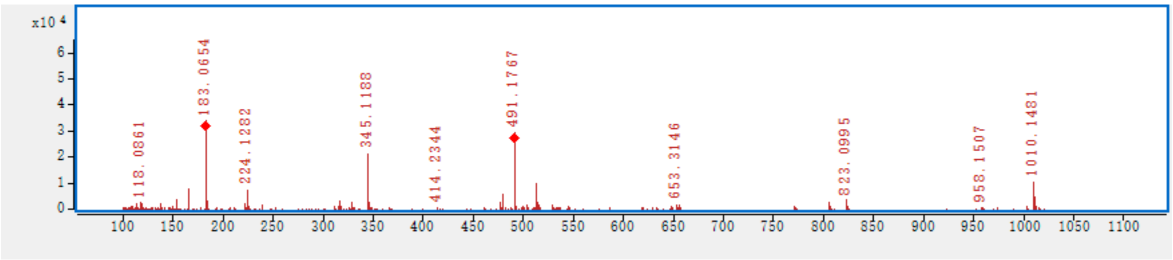

Figure S10 MS/MS spectrum of peak 5

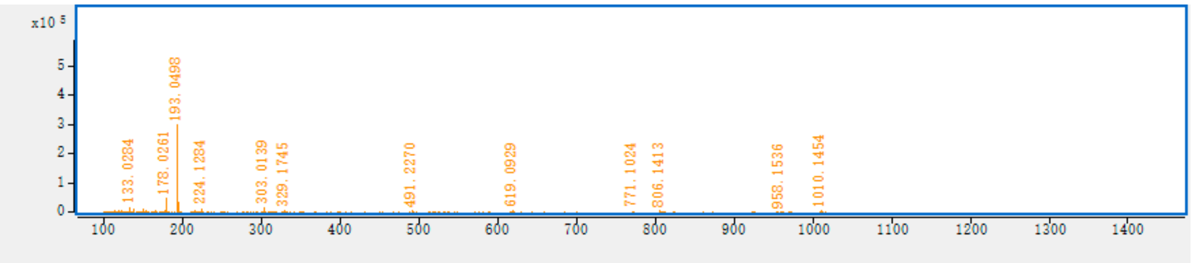

Figure S11 MS spectrum of peak 6

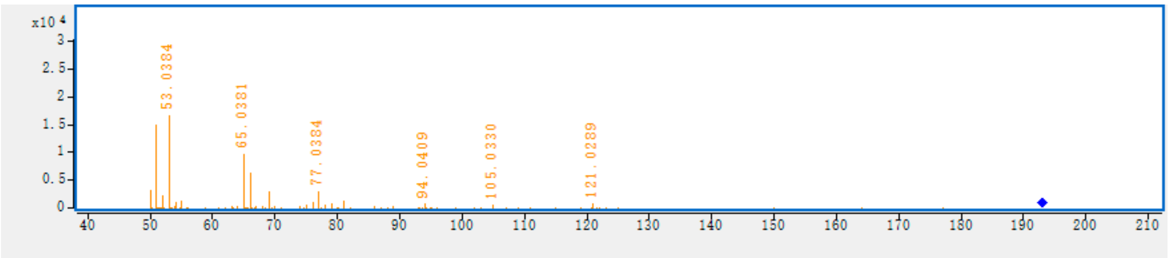

Figure S12 MS/MS spectrum of peak 6

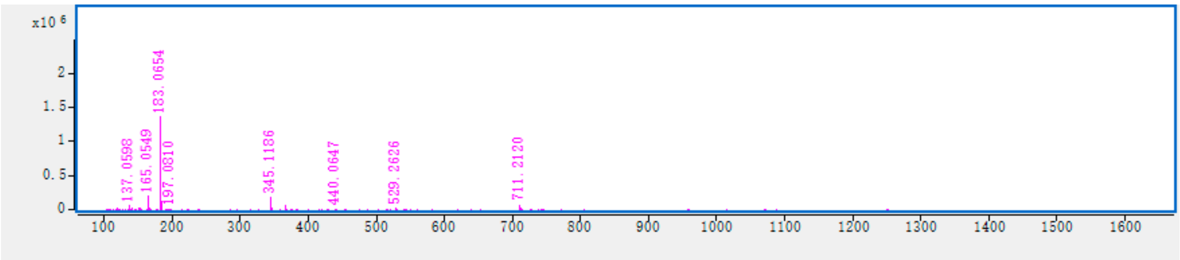

Figure S13 MS spectrum of peak 7

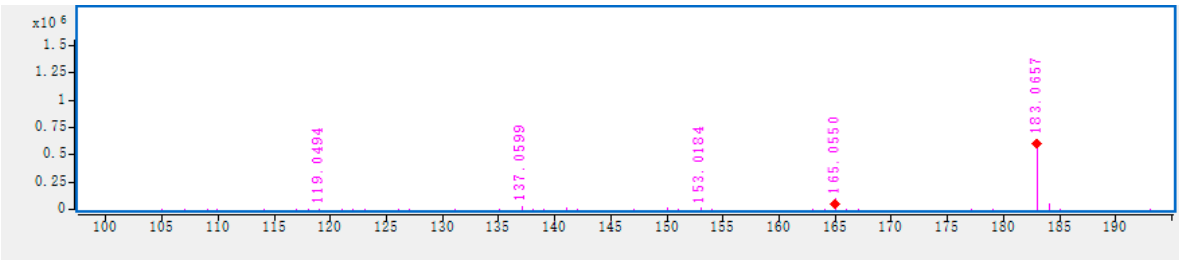

Figure S14 MS/MS spectrum of peak 7

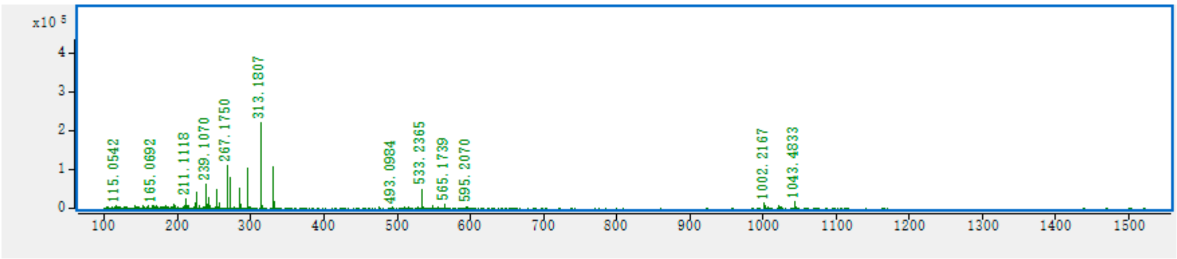

Figure S15 MS spectrum of peak 8

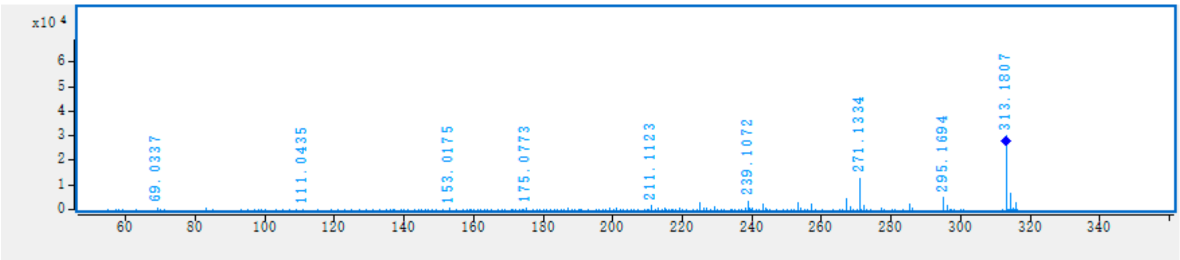

Figure S16 MS/MS spectrum of peak 8

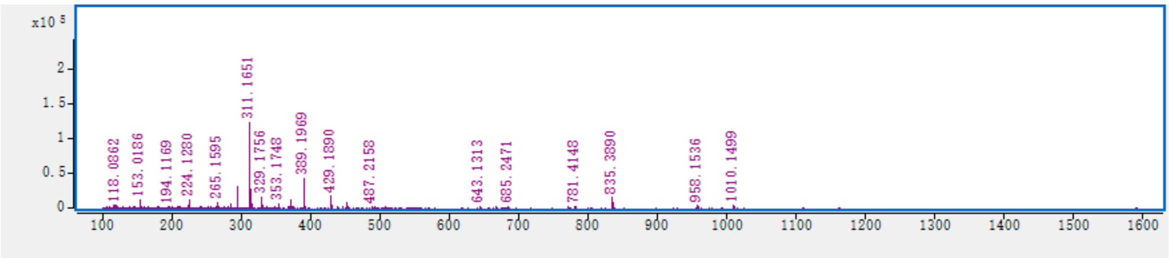

Figure S17 MS spectrum of peak 9

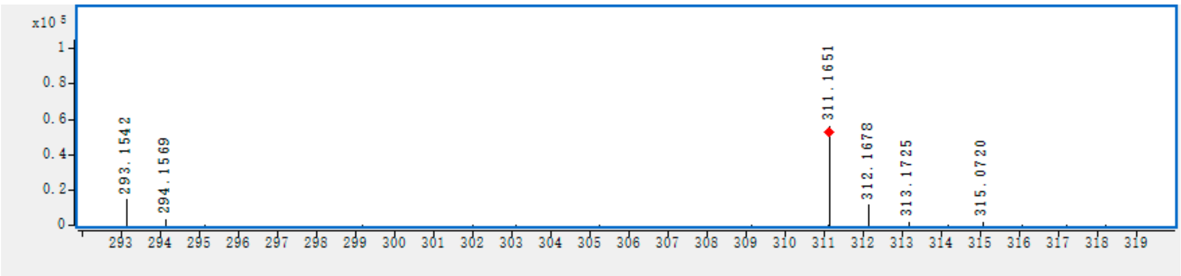

Figure S18 MS/MS spectrum of peak 9

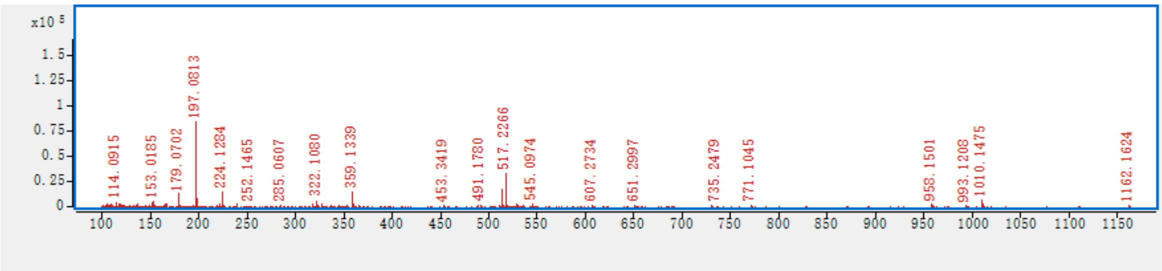

Figure S19 MS spectrum of peak 10

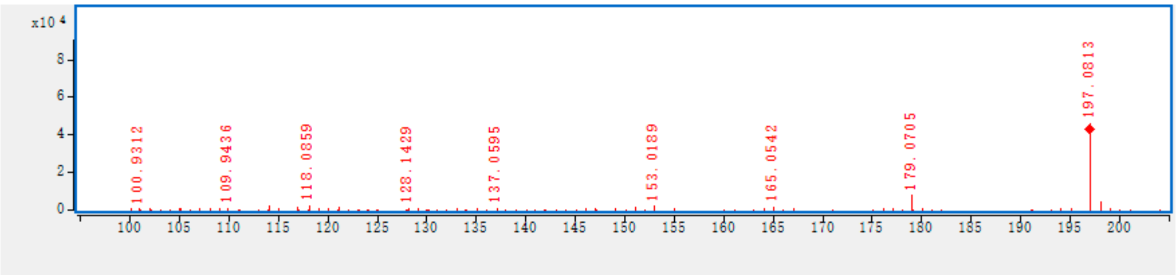

Figure S20 MS/MS spectrum of peak 10

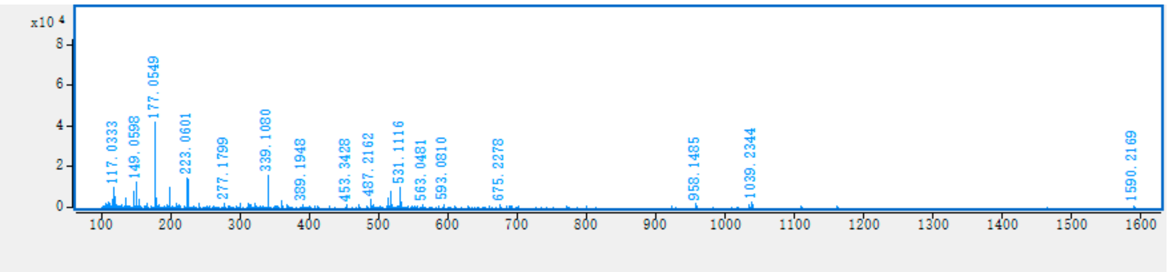

Figure S21 MS spectrum of peak 11

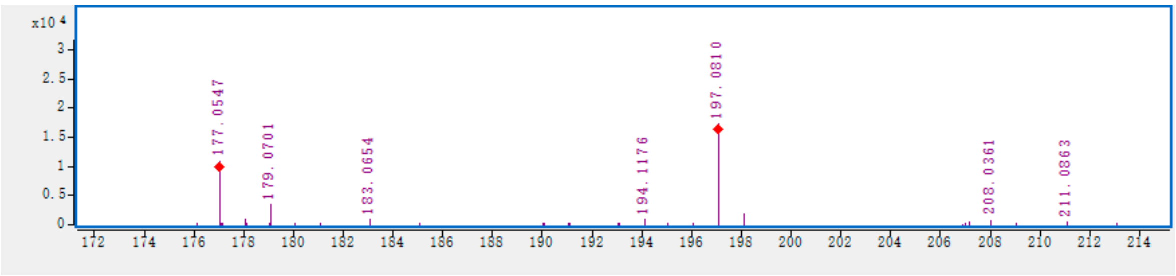

Figure S22 MS/MS spectrum of peak 11

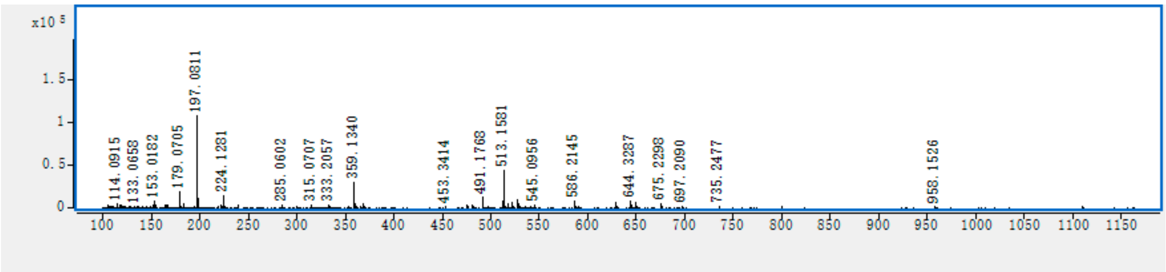

Figure S23 MS spectrum of peak 12

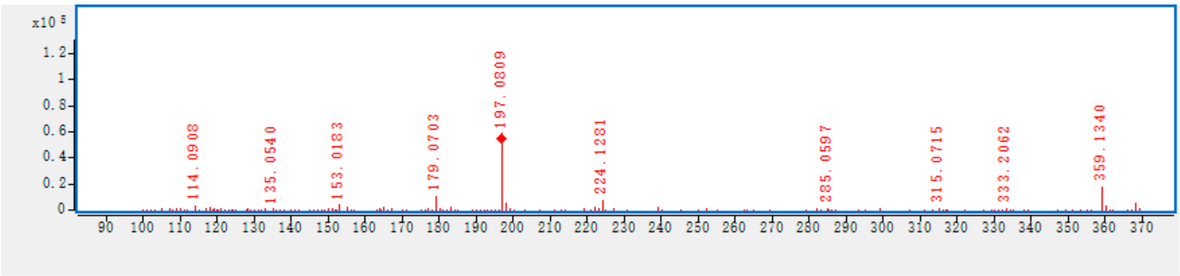

Figure S24 MS/MS spectrum of peak 12

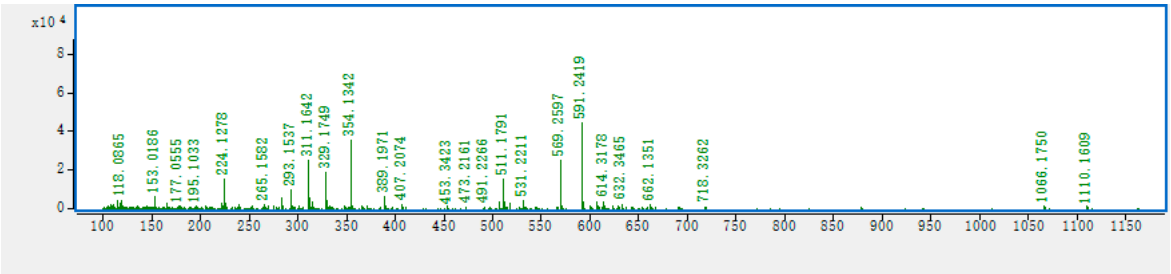

Figure S25 MS spectrum of peak 13

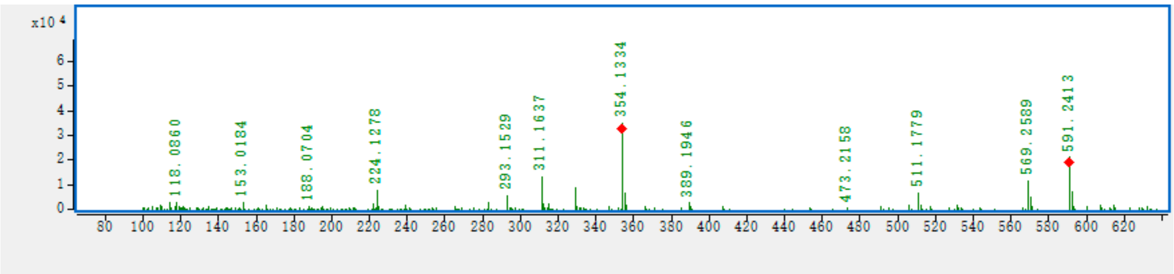

Figure S26 MS/MS spectrum of peak 13

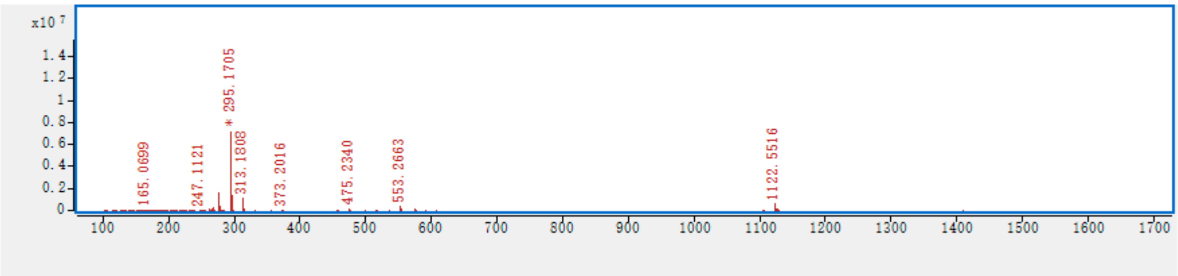

Figure S27 MS spectrum of peak 14

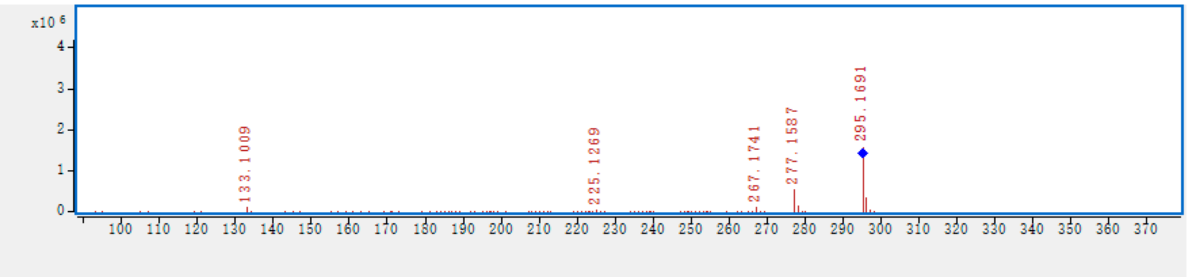

Figure S28 MS/MS spectrum of peak 14

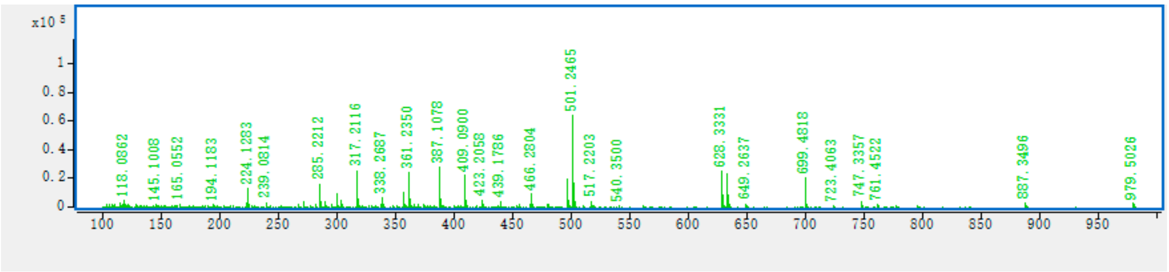

Figure S29 MS spectrum of peak 15

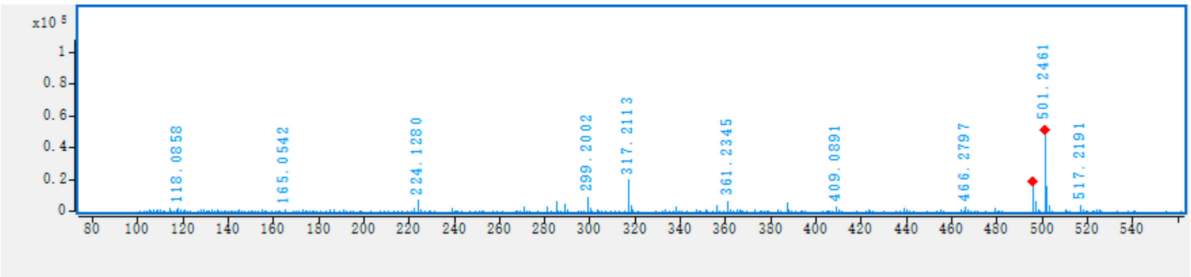

Figure S30 MS/MS spectrum of peak 15

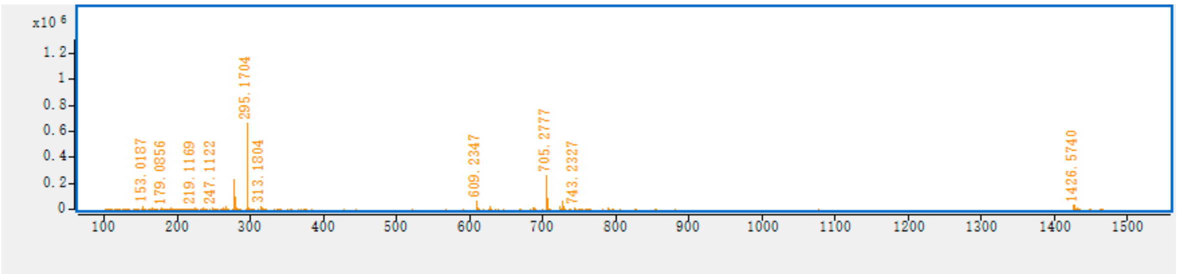

Figure S31 MS spectrum of peak 16

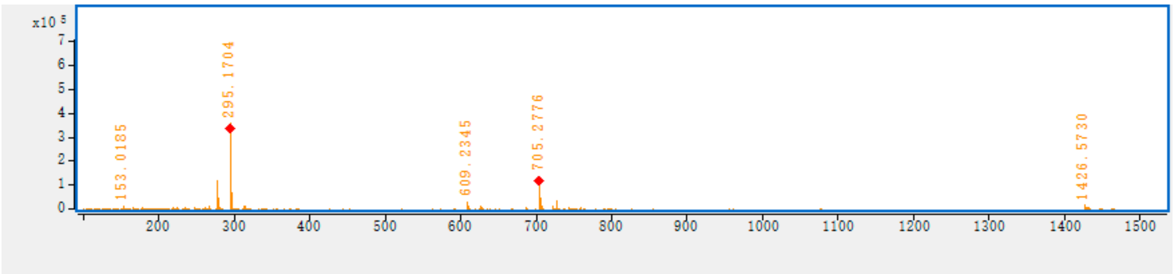

Figure S32 MS/MS spectrum of peak 16

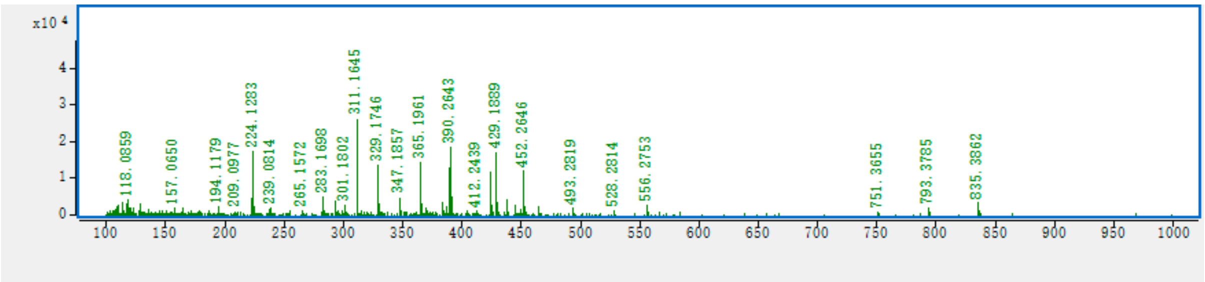

Figure S33 MS spectrum of peak 17

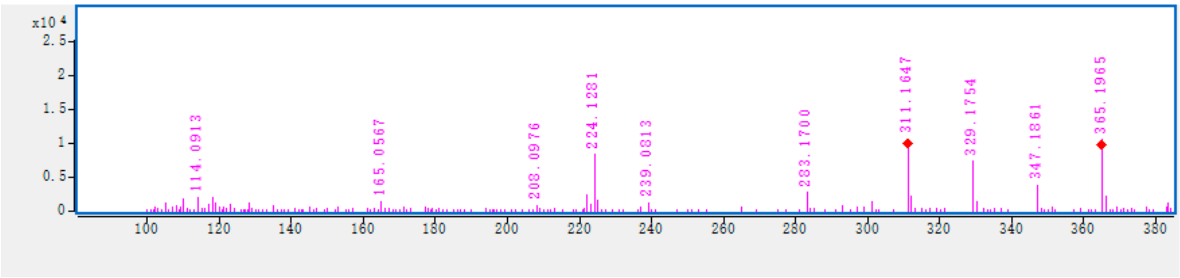

Figure S34 MS/MS spectrum of peak 17

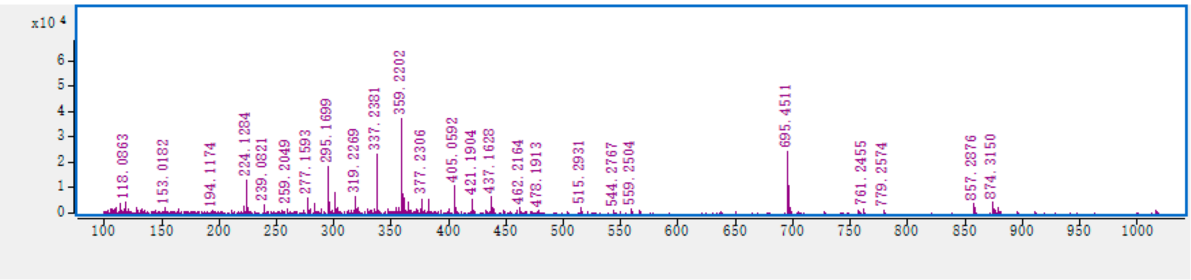

Figure S35 MS spectrum of peak 18

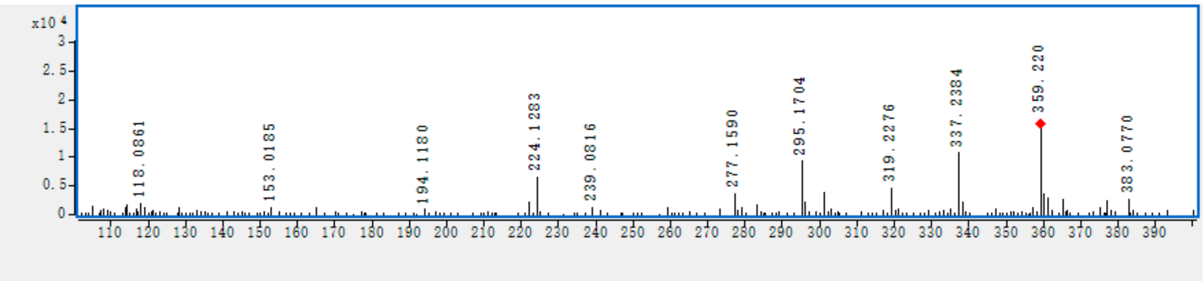

Figure S36 MS/MS spectrum of peak 18

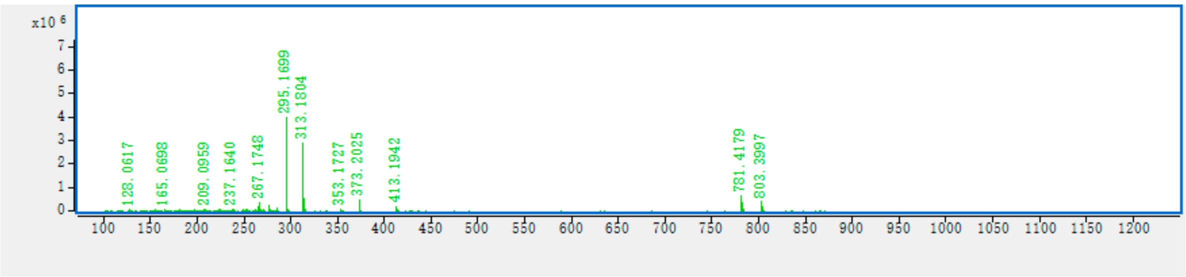

Figure S37 MS spectrum of peak 19

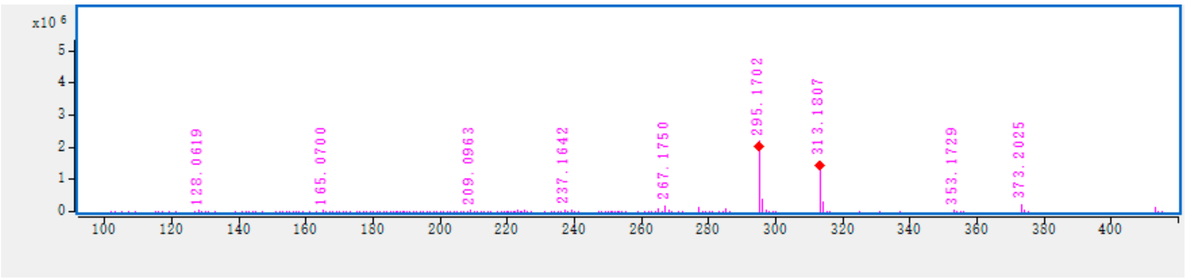

Figure S38 MS/MS spectrum of peak 19

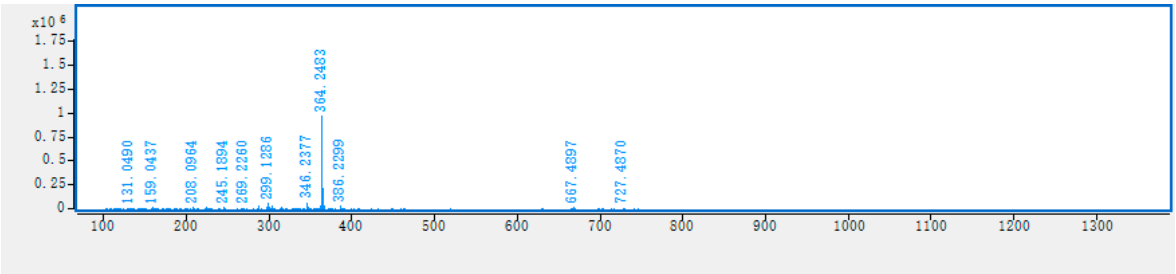

Figure S39 MS spectrum of peak 20

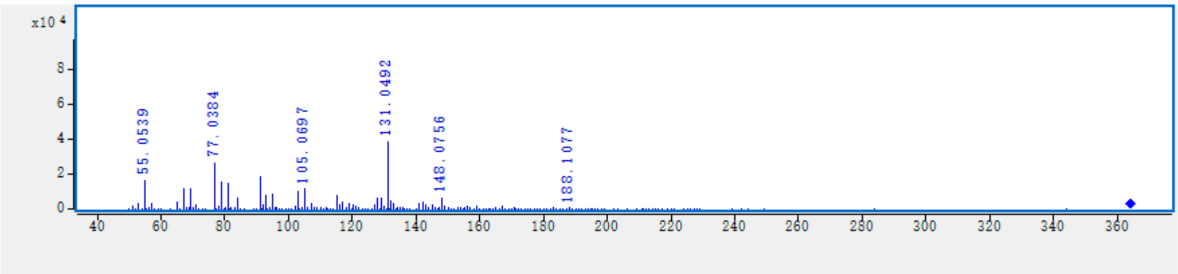

Figure S40 MS/MS spectrum of peak 20

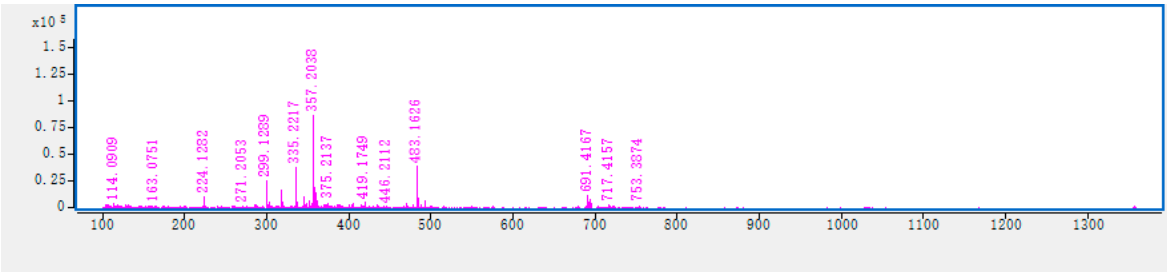

Figure S41 MS spectrum of peak 21

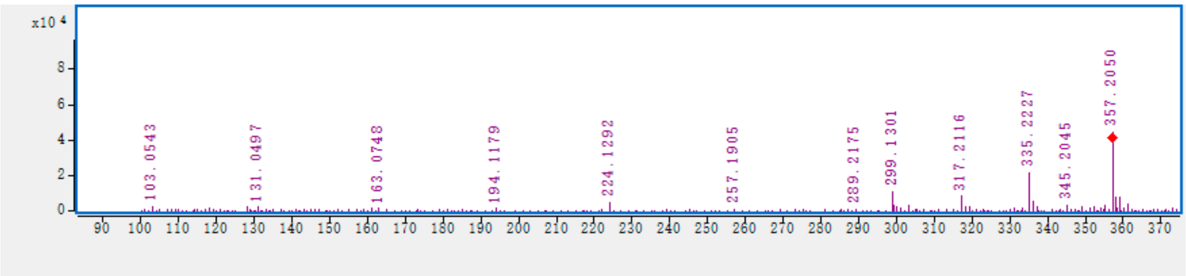

Figure S42 MS/MS spectrum of peak 21

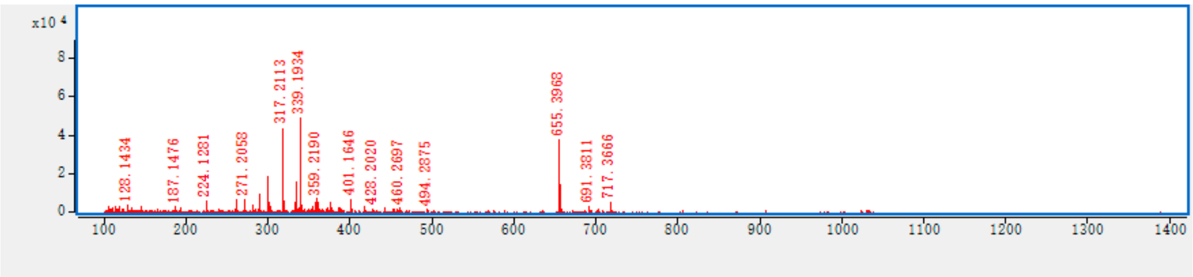

Figure S43 MS spectrum of peak 22

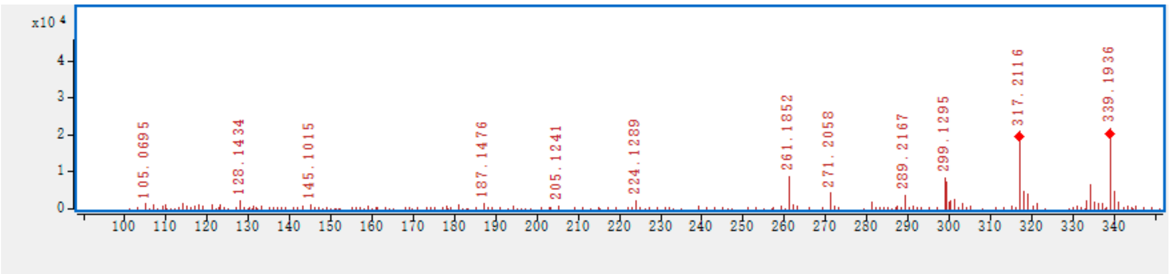

Figure S44 MS/MS spectrum of peak 22

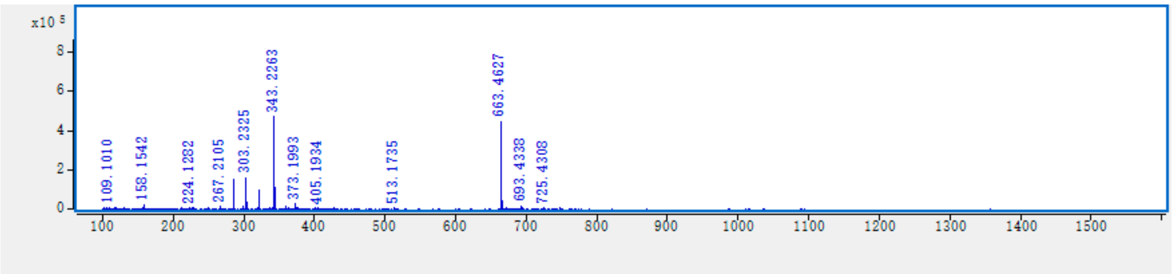

Figure S45 MS spectrum of peak 23

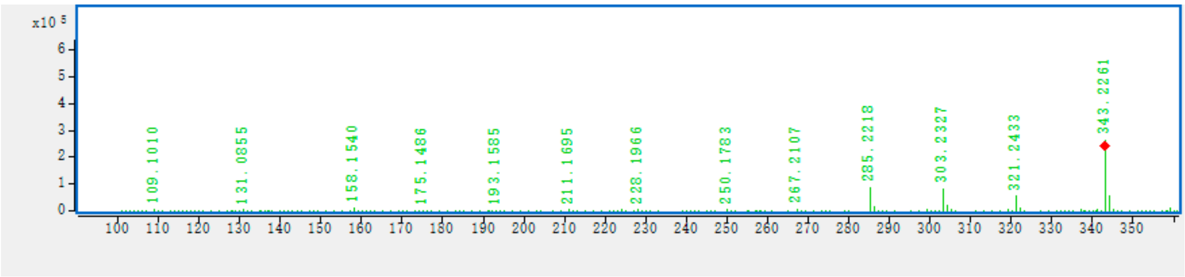

Figure S46 MS/MS spectrum of peak 23

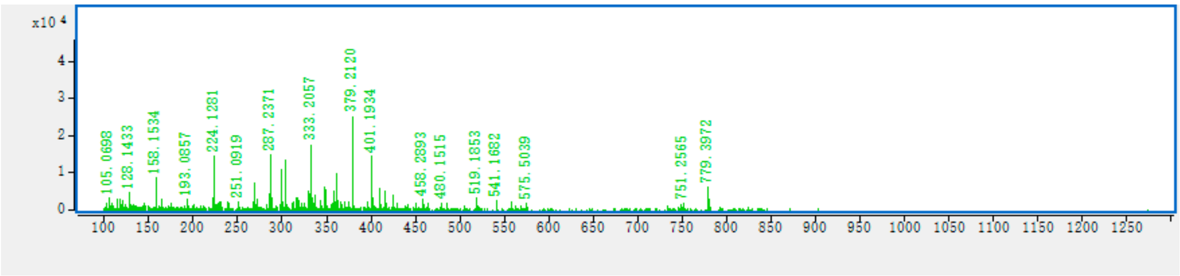

Figure S47 MS spectrum of peak 24

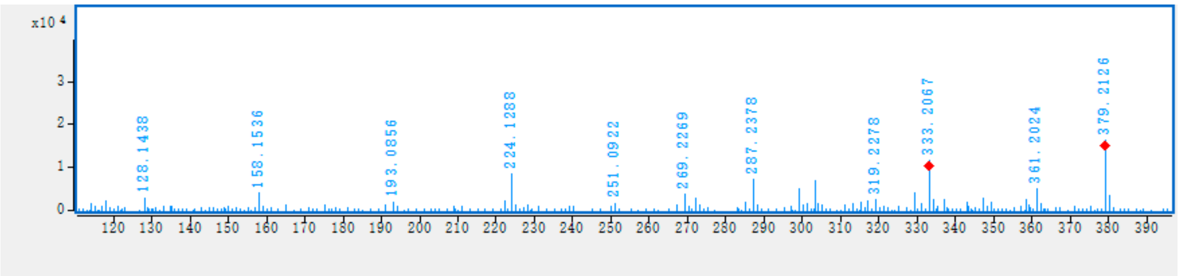

Figure S48 MS/MS spectrum of peak 24

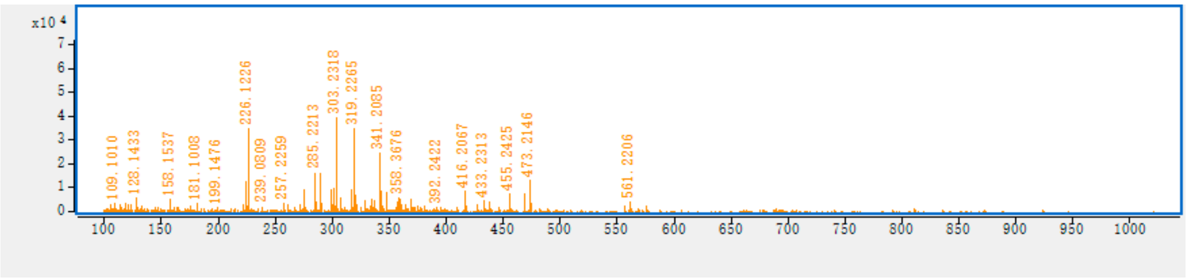

Figure S49 MS spectrum of peak 25

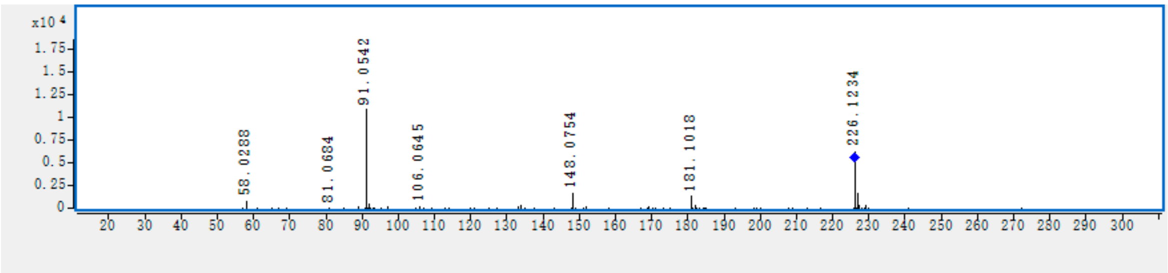

Figure S50 MS/MS spectrum of peak 25

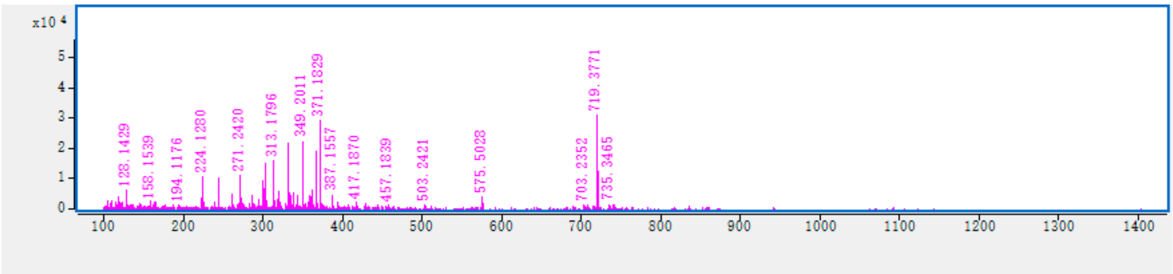

Figure S51 MS spectrum of peak 26

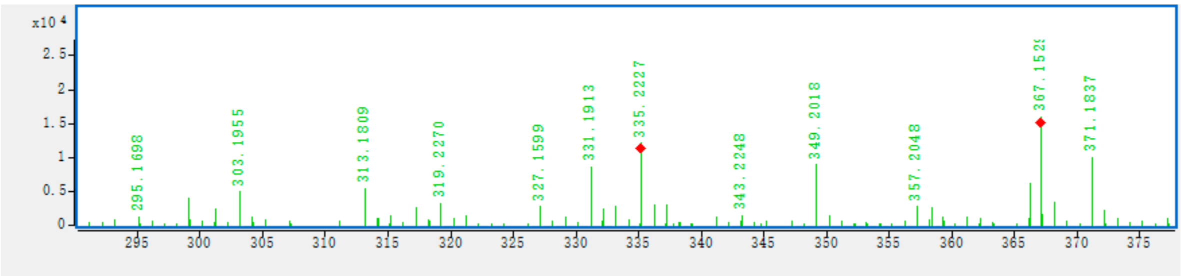

Figure S52 MS/MS spectrum of peak 26

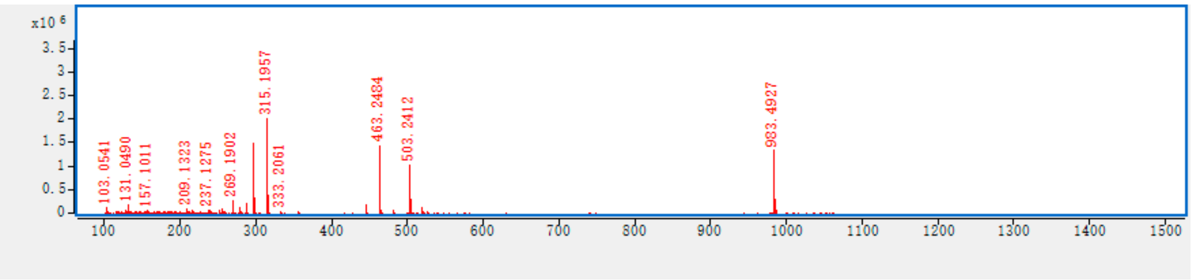

Figure S53 MS spectrum of peak 27

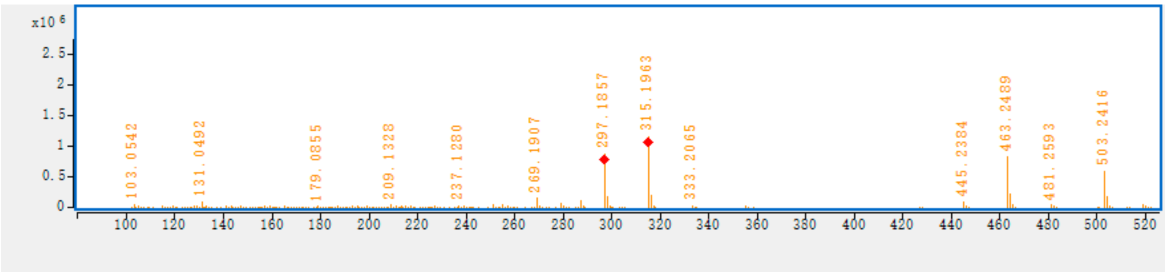

Figure S54　MS/MS spectrum of peak 27

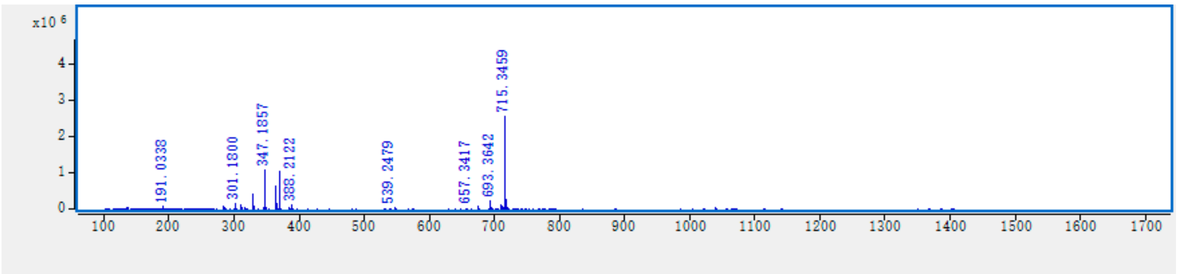

Figure S55　MS spectrum of peak 28

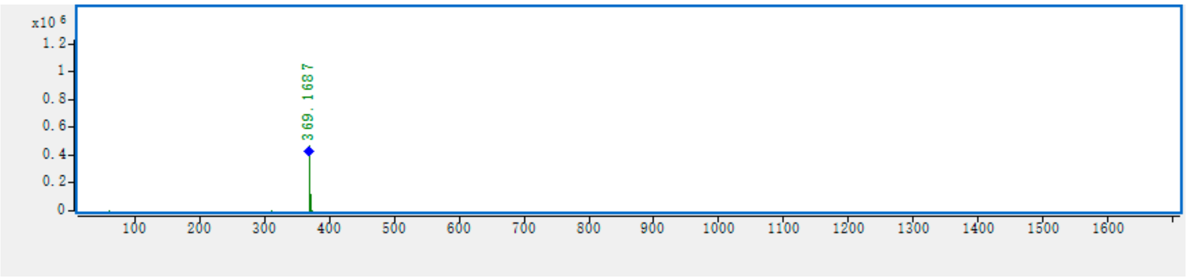

Figure S56　MS/MS spectrum of peak 28

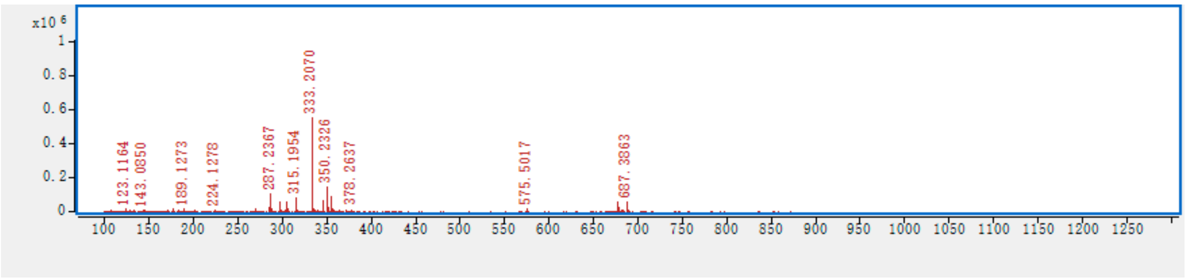

Figure S57　MS spectrum of peak 29

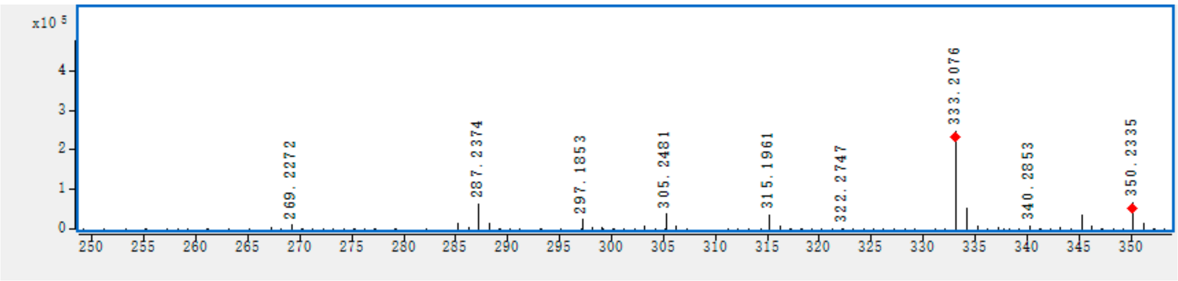

Figure S58　MS/MS spectrum of peak 29

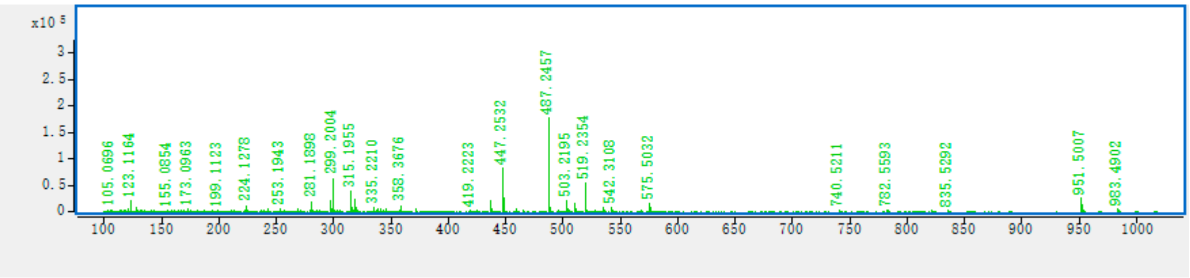

Figure S59 MS spectrum of peak 30

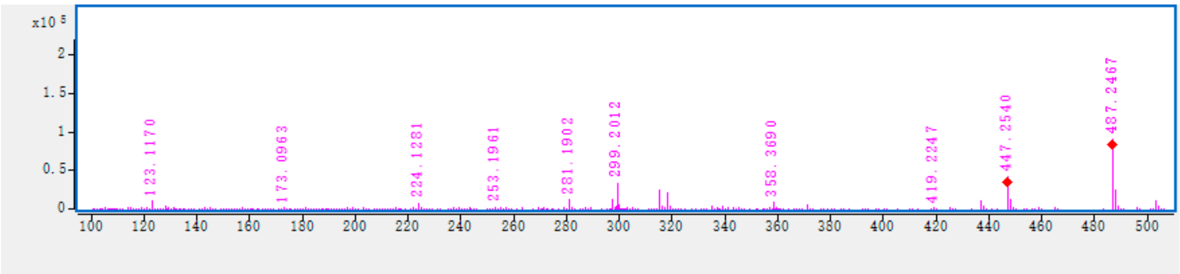

Figure S60 MS/MS spectrum of peak 30

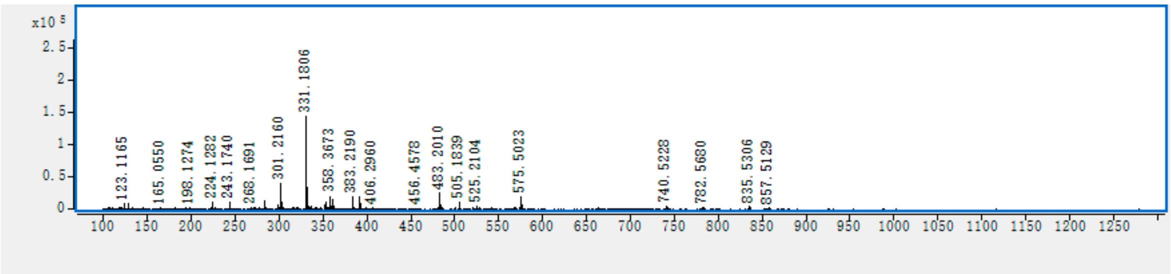

Figure S61 MS spectrum of peak 31

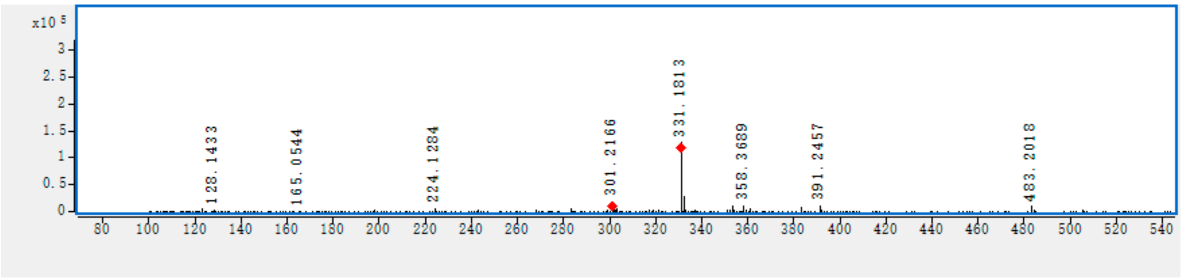

Figure S62 MS/MS spectrum of peak 31

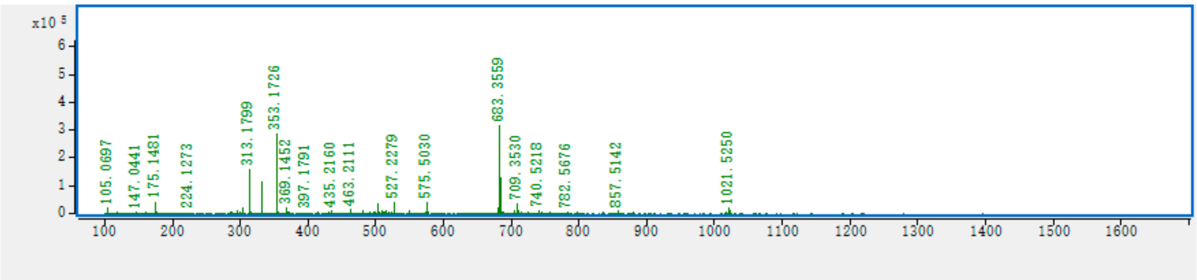

Figure S63 MS spectrum of peak 32

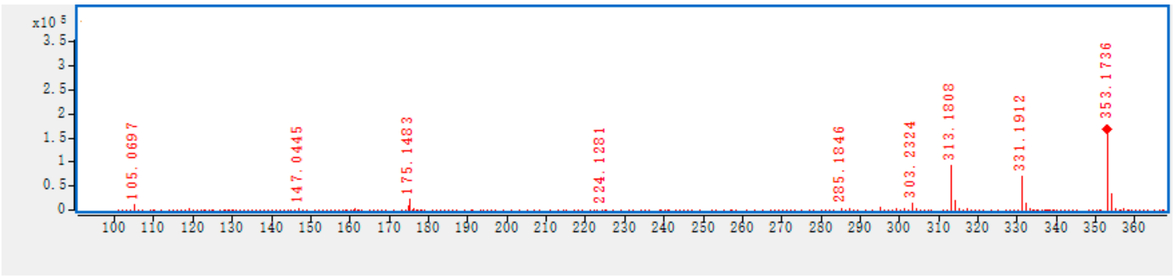

Figure S64 MS/MS spectrum of peak 32

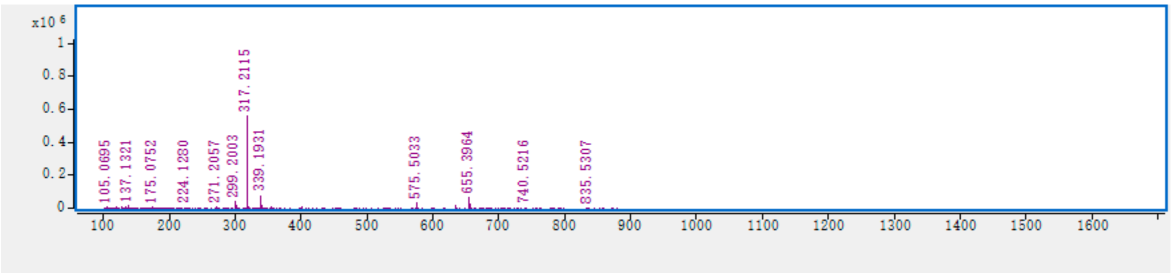

Figure S65 MS spectrum of peak 33

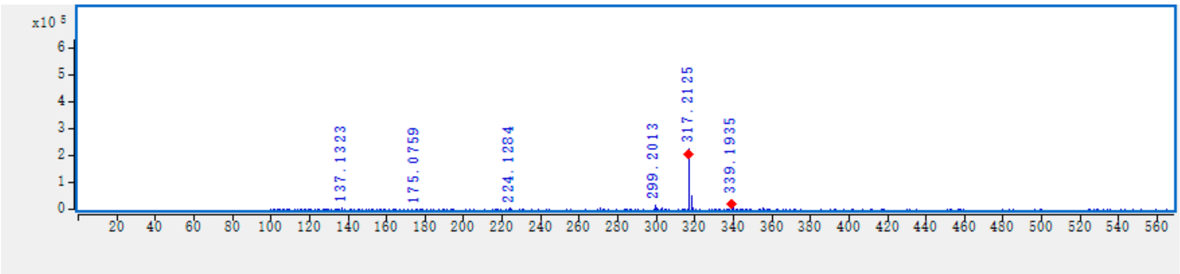

Figure S66 MS/MS spectrum of peak 33

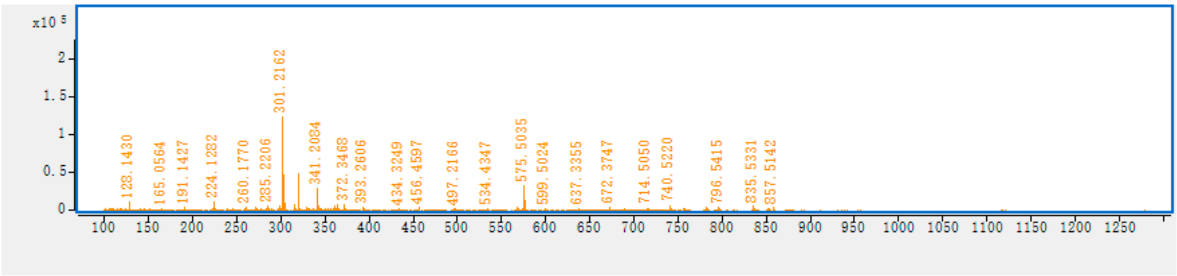

Figure S67 MS spectrum of peak 34

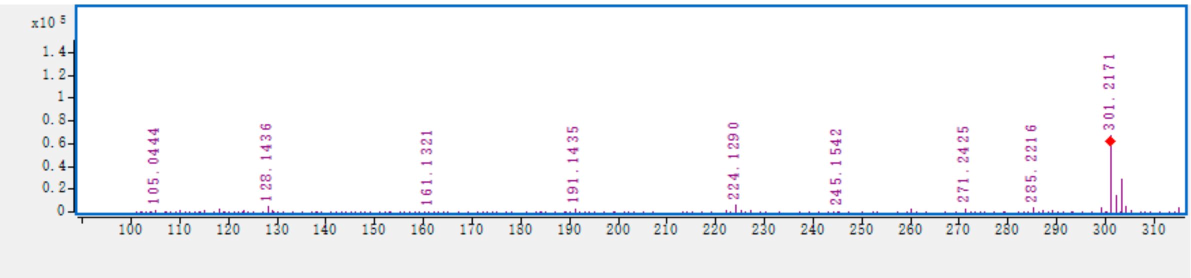

Figure S68 MS/MS spectrum of peak 34

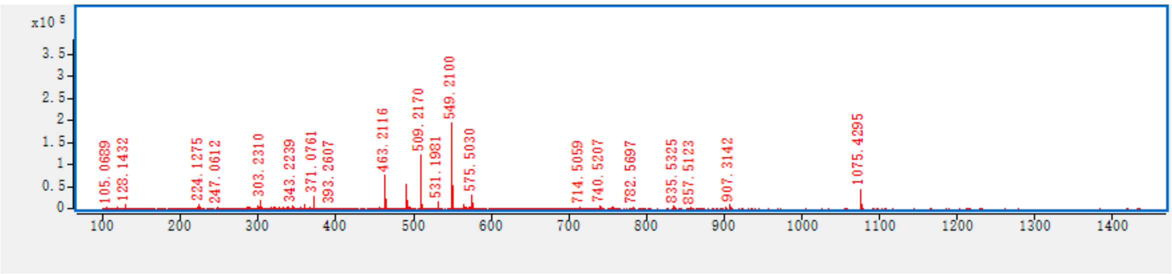

Figure S69 MS spectrum of peak 35

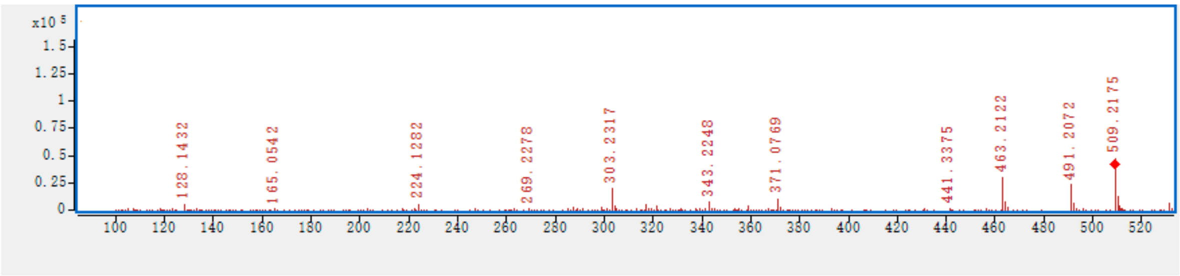

Figure S70 MS/MS spectrum of peak 35

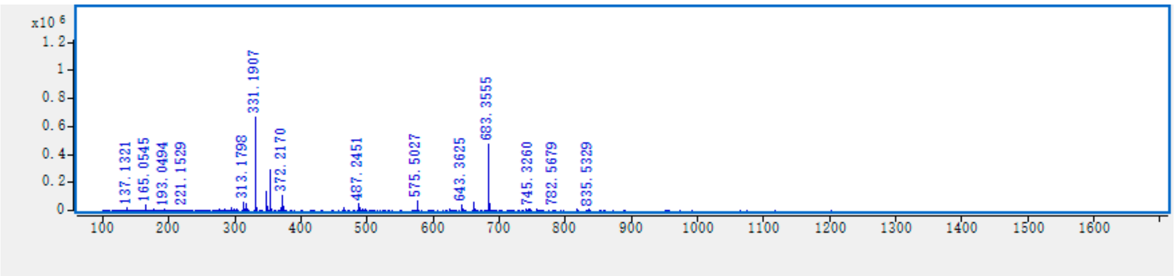

Figure S71 MS spectrum of peak 36

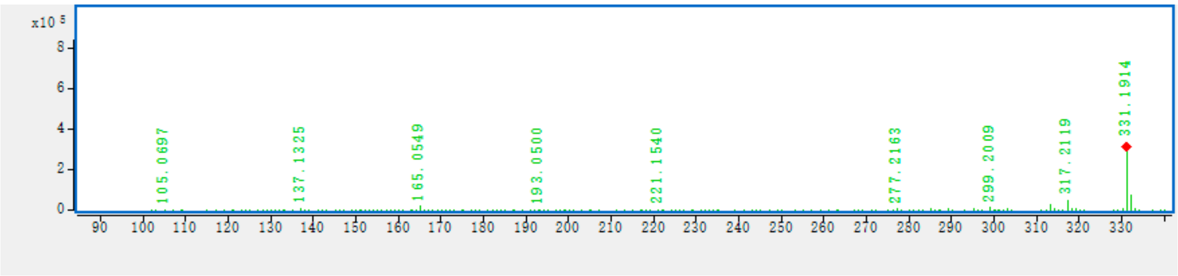

Figure S72 MS/MS spectrum of peak 36

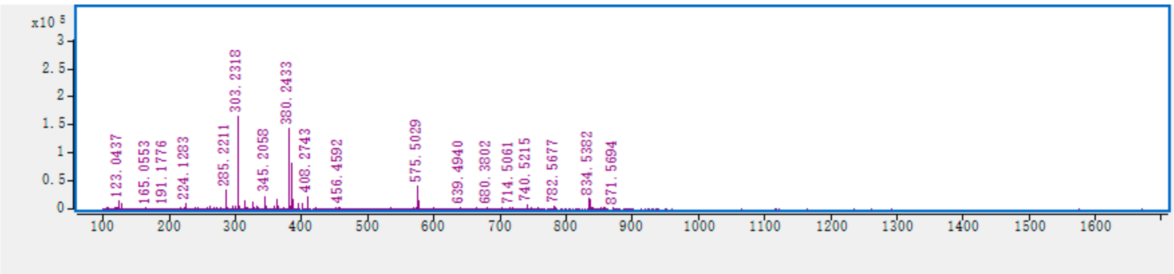

Figure S73 MS spectrum of peak 37

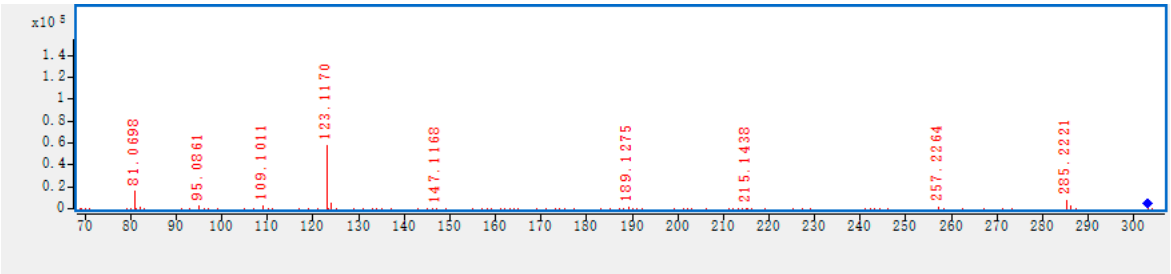

Figure S74 MS/MS spectrum of peak 37

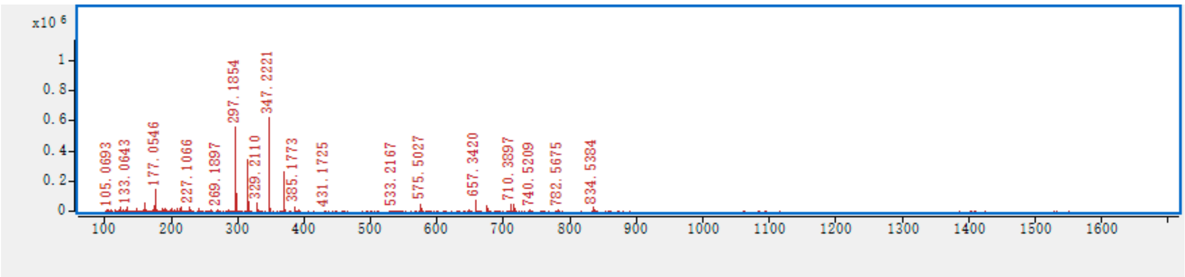

Figure S75 MS spectrum of peak 38

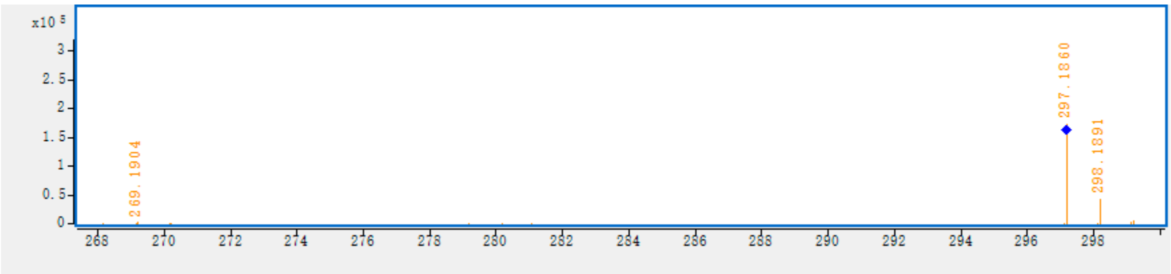

Figure S76 MS/MS spectrum of peak 38

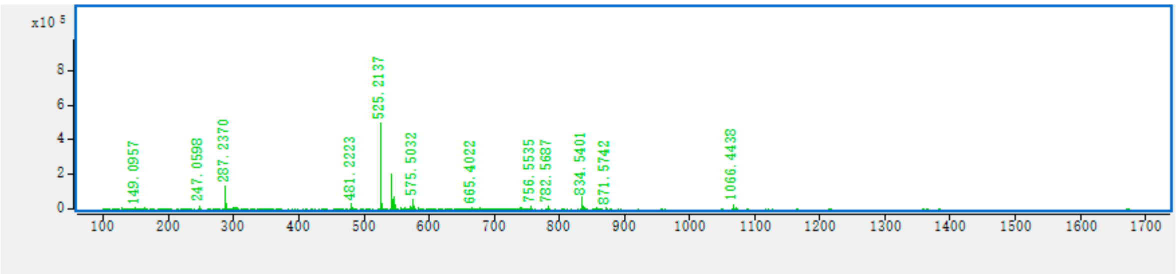

Figure S77 MS spectrum of peak 39

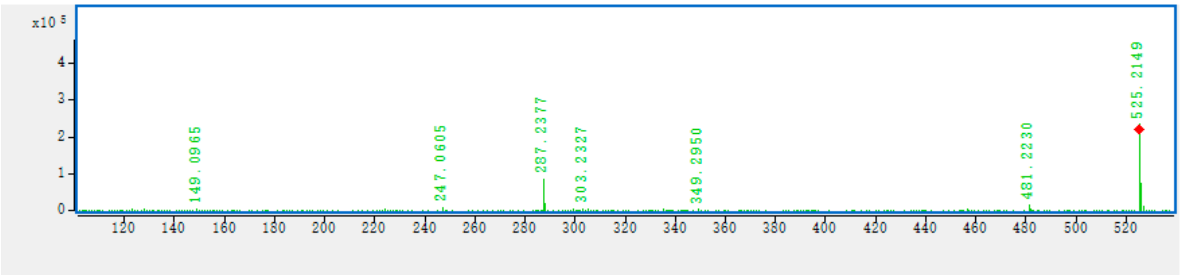

Figure S78 MS/MS spectrum of peak 39
